# CLC gene family in *Solanum lycopersicum*: genome-wide identification, expression, and evolutionary analysis of tomato in response to salinity and Cd stress

**DOI:** 10.3389/fpls.2025.1547723

**Published:** 2025-04-29

**Authors:** Jun Ma, Shan Li, Shah Zaman, Ali Anwar

**Affiliations:** ^1^ School of Grain and Food & Pharmacy, Jiangsu Vocational College of Finance and Economics, Huaian, Jiangsu, China; ^2^ School of Tea & Coffee, Pu’er University, Pu’er, China; ^3^ College of Horticulture, South China Agricultural University, Guangzhou, China

**Keywords:** *Solanum lycopersicum*, chloride channels, abiotic stress, alleviation of stress, anion transport, chloride ions

## Abstract

**Introduction:**

Chloride channels (CLCs) play critical roles in anion transport, stress adaptation, and ion homeostasis in plants. Whereas their genomic wide indentification and functional divergence in tomato (*Solanum lycopersicum*) remain largely unexplored.

**Methods and results:**

In this study, we identified nine CLC genes in the tomato genome, classifying them into two evolutionarily distinct clades (Group I and II) based on phylogenetic analysis. Structural dissection revealed conserved transmembrane domains (9–12 TMDs) and motif patterns (e.g., motifs 3/7/9 in Group I), with *SlCLC02* exhibiting the largest gene size (27,041 bp). Promoter analysis indicated the presence of key abiotic stress-responsive cis-elements (ABRE, MYB, MYC), aligning with the pronounced transcriptional dynamics of SlCLCs under salinity stress. Notably, qRT-PCR analysis demonstrated that most SlCLC genes (particularly *SlCLC05*, an ortholog to *AtCLC-g*) exhibited rapid upregulation within 1-4 hours followed by downregulation in roots under salinity treatment, suggesting early stress signaling roles. Likewise, preliminary expression profiling under cadmium stress further identified specific induction of *SlCLC07*, proposing gene-specific roles in heavy metal detoxification. Strikingly, *SlCLC09* lacked collinearity with Arabidopsis/potato homologs, implying lineage-specific diversification.

**Discussion:**

These findings elucidate the SlCLC family’s structural diversity, evolutionary constraints, and stress-responsive regulation, providing a framework for targeting specific SlCLC genes (e.g., *SlCLC05*) to enhance chloride homeostasis in crops under combined salinity and cadmium stress. This study will open a new research direction for genetic crop improvement to ensure protected vegetable production.

## 1 Introduction

World climate change, excessive irrigation requirements, and soil salinization have a substantial influence on current crop growth and production, limiting the sustainable expansion of irrigated agriculture and presenting a danger to world agricultural security (Hassani et al., 2021; Rehman et al., 2022; Muhammad et al., 2024). Sodium chloride (NaCl) is a predominant salinization agent in agricultural soils, imposing osmotic and ionic stress on crop plants (Zhou et al., 2024). While the toxic effects of sodium (Na^+^) on plants and the associated adaptive mechanisms have been extensively studied (Wu, 2018; Balasubramaniam et al., 2023), the role of chloride ions (Cl^−^) has been less explored. Although previous studies have suggested that chloride ions play an important role as micronutrients in plant growth, plants exhibit symptoms of nutrient deficiency when exposed to environments with low chloride ion concentrations (Broyer et al., 1954; Johnson et al., 1957). In addition, recent studies have proposed a new perspective on chloride ions as beneficial macronutrients. These studies suggest that chloride ions can improve plant water balance and water relations, promote avoidance and tolerance to water stress, and enhance drought resistance in plants (Franco-Navarro et al., 2016, 2021; Colmenero-Flores et al., 2019).

Soil salinity is typically characterized by the salt concentration as non-saline (less than 3 g/L), slightly saline (from 3 g/L to 6 g/L), medium saline (from 6 g/L to12 g/L), and highly saline (more than 12 g/L) (https://www.fao.org/4/r4082e/r4082e08.htm), and salinity in plant bulk tissues is typically expressed in terms of average Na^+^ and Cl^−^ content [mg/g dry weight, (DW)] or concentration (mmol/L, mM), considering that the Na^+^ and Cl^−^ content/concentration in plants varies among species without non-saline-treated soil. For example, in glycophyte plants, Cl^−^ is accumulated in tissues at concentrations 10 to 500 times higher than the micronutrient requirement (i.e., more than 50 mg/g DW compared to 0.2 mg/g DW), despite the high energy cost of uptake and accumulation (White and Broadley, 2001; Xu et al., 1999; Colmenero-Flores et al., 2019). For Na^+^, most major agricultural crops have poor tolerance to salinity. Although the concentration of sodium in tissues (approximately 1 g/kg DW) is closer to the range of micronutrients, at higher supply levels, the concentration of sodium in tissues approaches that of macronutrients, especially C4, in plant species. This may be related to its ability to substitute for potassium in osmotic regulation (Broadley et al., 2012). However, certain studies have demonstrated that, in a salinity scenario, the excessive accumulation of Cl^−^ in bulk plant tissues can result in several detrimental consequences for plant physiology (Almeida et al., 2014; Rajappa et al., 2024; Yang et al., 2023b), including leaf necrosis, impaired stomatal function, reduced photosynthetic activity, and an increase in the production of reactive oxygen species (Brumós et al., 2009; Wu and Li, 2019), leading to a cumulative suppression of plant growth and yield (Geilfus, 2018; Rajappa et al., 2024). Moreover, the efflux of Cl^−^ from the root system exhibits a positive correlation with plant salt tolerance (Geilfus, 2018), implicating this process as a potential principal mechanism to avert the toxic effects of chloride ions within plants. Some genes, such as the chloride channel protein family (CLC), cation–Cl^−^ cotransporter (CCCs), slow anion channel associated (SLAC/SLAH1), aluminum-activated malate transporter (ALMT), nitrate transporter/peptide transporter (NRT1/NPF), the multidrug and toxic compound extrusion (MATE), and the ion chloride nucleotide-sensitive protein (ICln1) family, are likely crucial for the nutrition, long-distance transport, and compartmentalization of chloride ions, and for regulating cell turgor and enhancing stress tolerance in plants (Brumós et al., 2010; Cubero-Font et al., 2016; Hedrich and Geiger, 2017; Liu et al., 2024; Wu et al., 2025).

CLCs were initially discovered in the electric ray *Torpedo californica* (Miller and White, 1980), but the first found in tobacco (*Nicotiana tabacum*) plants was *Nt*CLC1 (Lurin et al., 1996; Zhang et al., 2018). The function of the CLCs has been confirmed in several species, with seven CLC members in *Arabidopsis thaliana* (Nedelyaeva et al., 2020), eight CLC members in soybean (Wei et al., 2019; Liu et al., 2021), 17 CLC genes in tobacco (Zhang et al., 2018), and 22 CLC genes in *Brassica napus* (Liu et al., 2020) identified (Liu et al., 2022). They were identified as an anion channel or transporter, controlling intracellular organelle ion homeostasis and acidification by moving anions across the membrane, which is generally separated into anion channels and anion/proton antiporters (Yang et al., 2023a). A more comprehensive analysis revealed that CLCs, as intrinsic membrane proteins, have a structure consisting of 10 to 12 transmembrane domains (Dutzler et al., 2002; Wei et al., 2019), and play an important role in vesicular transport mainly by regulating the internal pH, such as alkalinization in tobacco endoplasmic reticulum (EPR) lume, or the transmembrane electric potential (Δψ) of organelles (von der Fecht-Bartenbach et al., 2007; Sun et al., 2018). However, the hypotheses formulated from the existing data require further exploration. Some reports suggested that CLCs were present in the cell membranes and involved in various cellular functions (Jentsch et al., 1990; Jentsch, 2008). Moreover, many reports revealed that CLCs are located in the endomembrane system, vacuolar membranes (*At*CLCa, *At*CLCc, *At*CLCg, *Gm*CLC1, and *Th*CLC-a) (Li et al., 2006; Jossier et al., 2010; Yang et al., 2013, 2023b; Zhou et al., 2013; Wege et al., 2014; Nguyen et al., 2016; Liu et al., 2022; Balasubramaniam et al., 2023; Hodin et al., 2023), Golgi vesicles (*At*CLCd and *At*CLCf) (von der Fecht-Bartenbach et al., 2007; Marmagne et al., 2007; Guo et al., 2014; Scholl et al., 2021), thylakoid membrane of chloroplasts (*At*CLCe), mesophyll cells (*At*CLCa) (Geelen et al., 2000; De Angeli et al., 2006), guard cells (*At*CLCa) (Wege et al., 2014; Hodin et al., 2023), and mitochondria (*Zm*CLCc) (Tampieri et al., 2011). Although some genes, such as *OsCLC1* and *CsCLCs*, have already been studied, their functions remain to be established (Diédhiou and Golldack, 2006; Colmenero-Flores et al., 2007; Brumós et al., 2009, 2010; Migocka et al., 2013; Um et al., 2018; Liu et al., 2024). These research studies revealed that CLCs mediate the uptake and translocation of anions such as Cl^−^, ensuring anion homeostasis (Dutzler et al., 2002; Wei et al., 2019), play a role in nutrient transport (Ludewig et al., 1996), and contribute to modulating membrane potential, maintaining turgor pressure (Dutzler et al., 2002) and controlling stomatal movement (De Angeli et al., 2013; Yang et al., 2023a). Furthermore, they are involved in hormone signaling (Barbier-Brygoo et al., 2011), embryonic development, and adapting to various abiotic and biotic stresses (Jossier et al., 2010; Nedelyaeva et al., 2020, 2022).

It has been revealed that CLCs serve as pivotal regulators in the maintenance of Cl^−^ equilibrium under saline conditions (Liu et al., 2021). In soybean plants, *GsCLC-c2* has been reported to play a role in regulating anionic homeostasis and maintaining lower Cl^−^/NO_3_^−^ ratios in shoots to enhance NaCl tolerance (Liu et al., 2021). Recently, Rajappa et al. (2024) found the NaCl-induced translocation of *At*CLCf to the plasma membrane (PM) from the Golgi in *Arabidopsis thaliana*, thus increasing the efflux of Cl^−^ from the root system and plant tolerance to saline conditions. In addition to enhancing efflux, CLCs also play a role in ion sequestration, such as *Gm*CLC1 and *Os*CLC-1 (Nakamura et al., 2006), vacuolar-located ion transporters that sequester ions from the cytoplasm into the vacuole, thereby mitigating their toxic effects (Li et al., 2006), or *At*CLCd, first transported into the trans-Golgi network and related compartments, and then transferred from the cytoplasm to the vacuole or apoplast by the endosomes (von der Fecht-Bartenbach et al., 2007). Several studies reported that CLCs are regulated by a variety of factors. As for *At*CLCa, its activity is regulated by nucleotides and phospholipids (Jentsch and Pusch, 2018), allowing them to sense the ATP/AMP ratio and modulate *At*CLCa accordingly, binding with PI(4,5)P2 or PI(3,5)P2 in the protein dimeric interface and occupying the proton exit pathway to promote vacuolar acidification and stomatal closure (Yang et al., 2023a). The *Zm*CLCg protein was initially proved to be associated with salt tolerance in maize (Luo et al., 2021). Interestingly, a response regulator *Zm*RR1 has also been proven to have the same function and can modulate Cl^−^ exclusion from shoots but the potential regulatory relationship between *Zm*RR1 and CLCs in maize is yet to be reported. These findings highlight the importance of CLC functions in plant salt tolerance, however, the underlying molecular mechanisms remain unclear. The collected evidence points to a model where the Cl^−^/H^+^-antiporter, Na^+^/H^+^-antiporter, and V-type H^+^-ATPase work in concert within endosomes.

Tomato (*Solanum lycopersicum* L.), a moderately tolerant species to salinity, is a significant horticultural crop with economic and nutritional importance (Sofy et al., 2021). It is vulnerable to salt stress, and excessive salinity levels influence seed germination, plant growth, and fruit development (Roşca et al., 2023). Research has identified specific genes associated with Cl^−^ transport in tomato plants, contributing to the understanding of salt tolerance in this species; however, studies focusing on tomato CLC genes remain limited. Bioinformatics and experimentation have significantly enhanced the understanding of the structure, function, and regulatory expression of CLC proteins. The precise role of CLC proteins in tomatoes under saline conditions remains unclear, despite a noticeable association with salt stress. This study seeks to elucidate the mechanisms by which CLCs function in tomatoes and evaluate their role in salt stress resistance, which is crucial for theoretical understanding and practical applications in agricultural plant breeding.

## 2 Materials and methods

### 2.1 Identification and sequence of the CLC gene family in tomato

The latest genomic data of tomato (ITAG5.0) were obtained from Phytozome13 (https://phytozome-next.jgi.doe.gov). We only preserved the longest protein sequence of each gene to eliminate redundancy. The identified CLC protein sequences from *Arabidopsis* (https://www.arabidopsis.org/), wheat, soybean, and potato were downloaded. These sequences were queried using the BLAST to retrieve homology genes from the tomato genome with E-value of 10^-5^. The CLC domain (PF00654) from the Pfam database in InterPro (https://www.ebi.ac.uk/interpro/) was employed as a query searched by Hidden Markov model (HMM) profiles with an E-value of 10^-5^. Furthermore, the protein sequences identified by both the above methods in the *S. lycopersicum* genome were integrated. The remaining proteins were considered candidate tomato CLC proteins. We submitted all candidate sequences to NCBI-CDD (https://www.ncbi.nlm.nih.gov/cdd/) and MEME to verify the CLC conserved domains and motifs (Bailey et al., 2009).

The molecular weight (MW), isoelectric point (pI), instability index (II), aliphatic index (AI), and grand average of hydropathicity (GRAVY) of these identified proteins were investigated using ExPASy (http://web.expasy.org/protparam/) online software. Subcellular localization of them were predicted based on the WolfPSORT (https://wolfpsort.hgc.jp/).

### 2.2 Phylogenetic relationship, gene structure, and conserved motifs analysis

To understand the evolutionary relationship of the tomato CLC genes, multiple sequence alignments of the identified CLC proteins of *Arabidopsis*, wheat, and potato, were performed using Muscle. A neighbor-joining (NJ) tree was constructed using IQ-tree software with a bootstrap value of 1,000. The chromosome physical location of the CLC genes was displayed using the Gene Location Visualize function of TBtools (Gasteiger et al., 2005; Chen et al., 2020).

### 2.3 Analysis of cis-acting elements in *CLC* promoters

The PlantCARE online tool (http://bioinformatics.psb.ugent.be/webtools/plantcare/html/) was used to analyze the 2 kb upstream sequence of the promoter sequence from the tomato *CLC* family gene (Rombauts et al., 1999).

### 2.4 Chromosome distribution and collinearity analysis

The positional information and chromosome lengths of *SlCLC* gene members were from a ITAG 5.0 gff file. These data were compared and visualized through covariance analysis using the Multiple Co-linear Scanning Toolkit (MCScanX) (Wang et al., 2012).

### 2.5 Protein tertiary structure and gene expression pattern analysis

CLC proteins are integral in facilitating the transmembrane transport of anions. Within this gene family, SwissModel (https://swissmodel.expasy.org) was employed to visualize the 3D structure with rainbow color (Waterhouse et al., 2018). The online TomExpress platform (https://tomexpress.gbfwebtools.fr/query) and relevant data mining tools were used to carry out comprehensive transcriptomic profiling of eight *SlCLC* genes in tomato vegetative and reproductive tissues (Zouine et al., 2017).

### 2.6 Plant materials, NaCl and Cd treatments, RNA extraction, qRT-PCR, and expression of *Sl*CLCs in tomato tissues

Tomato was used for expression analysis under salt (NaCl) and Cd stress. Tomato seeds were cultured in soils for 21 days with one seedling in one pot with 16 h light (350 μmol m^−2^ s^−1^ light intensity, 28°C) and 8h darkness (28°C) in a plant growth room in Guangzhou, China. The 21-day seedlings were cultivated and watered with 20 mL supplemented with 100 μM Cd or 100 mM NaCl (China National Pharmaceutical Group Co., Ltd.) for 0 h (CK), 1 h, 2 h, 4 h, 8 h, 12 h, or 24 h in one pot. The leaves were sampled, quickly frozen in liquid nitrogen, and stored at -80°C. Each treatment was independently replicated three times. A TianGen RNA Plant Kit was used to extract total RNA. qRT-PCR was performed on a LightCycler 96 (Roche). The 10 μL reaction volume contained 5 μL of 2×SYBR Green Mix, 2 μL of cDNA, 0.5 μL of forward and reverse primers (**Supplementary Table S6**), and 2 μL double distilled water (ddH_2_O). SlACT was used as a reference gene. The results were calculated using the 2^−ΔΔCt^ method (Livak and Schmittgen, 2001). The data are presented as the mean ± standard error of the mean (SEM). Statistical analysis was performed using GraphPad Prism 9.0 software, employing one-way analysis of variance (ANOVA).

## 3 Results

### 3.1 Identification and phylogenetic analysis of the *SlCLC* family members in tomato

**Figure 1:**
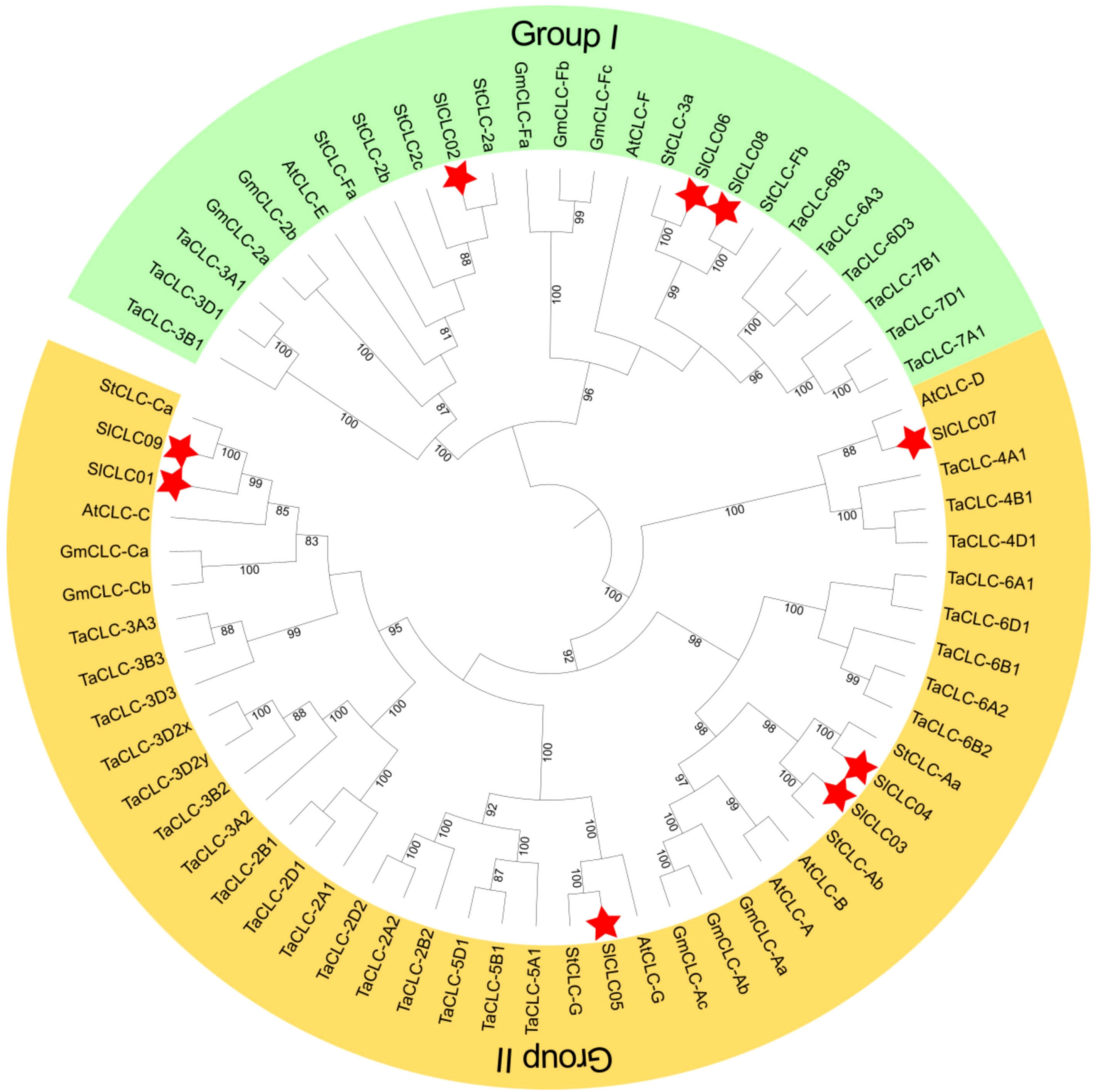
Phylogenetic analysis of evolutionary relationships of CLCs in *Arabidopsis* (*Arabidopsis thaliana*, AtCLC), soybean (*Glycine max*, GmCLC), potato (*Solanum tuberosum*, StCLC), wheat (*Triticum aestivum* L., TaCLC), and tomato (*Solanum lycopersicum* L. SlCLC). Green and yellow indicate Groups I and II. A red star represents tomato.

Nine genes, designated *SlCLC01* to *SlCLC09*, were identified through BLASTP and HMM searches of the tomato genome, considering homology, conserved domains, and transmembrane domains. However, the gene length, CDS length of *SlCLC01*, and its amino acid count were unusually extensive, measuring 20,890, 5,262, and 1,753 bp, respectively (**Supplementary Tables S1**, **S2**). Therefore, we checked it again using Fgenesh software (http://www.softberry.com/cgi-bin/programs/gfind/fgenesh.pl), and found it to be related to two genes, *SlCLC_6_*like and PH-like superfamily (cl17171) (**Supplementary Figure S1**, **Supplementary Table S3**). Therefore, we reconstructed it with shorter genes, only preserving the *SlCLC_6_*like gene, and named it *SlCLC01* for phylogenetic analysis. The corresponding amino acid sequences were subjected to bioinformatics analysis, including an assessment of their physicochemical properties. The results showed that the length of *SlCLC* genes varied from 27,041 bp (*SlCLC02*) to 3,068 bp (*SlCLC04*), with CDS lengths from 2,223 bp (*SlCLC02*) to 2,538 bp (*SlCLC09*). The amino acid number of *SlCLC* genes varied from 740 bp (*SlCLC02*) to 845 bp (*SlCLC09*). The largest protein had a molecular weight of 191.14 kDa (*SlCLC01*), while the smallest protein weighed 79.24 kDa (*SlCLC02*), with an average molecular weight of 97.48 kDa. Among the *SlCLC* family members, three proteins (*Sl*CLC02, *Sl*CLC06, and *Sl*CLC08) of the Group I members were considered acidic (pI < 7), while six proteins (*Sl*CLC01, *Sl*CLC03, *Sl*CLC04, *Sl*CLC05, *Sl*CLC07, and *Sl*CLC09) of the Group II members were classified as basic (pI > 7) (**Table 1**; **Figure 1**). The isoelectric points of the proteins fell within the range of 6.42 to 8.82. The instability index in the *Sl*CLC proteins varied from 33.23 to 48.41. The aliphatic index and GAH of *Sl*CLC proteins ranged from 94.14 (*Sl*CLC01) to 113.42 (*Sl*CLC04) and from -0.08 (*Sl*CLC01) to 0.37 (*Sl*CLC05), respectively.

**Table 1:** Physicochemical property characterization of the CLC gene family identified in tomato.

ID	NAA	MW (kDa)	PI	II	AI	GAH	PSL	TMDs
*SlCLC01*	1753	191,139.97	8.79	42.08	94.14	-0.08	Chloroplast	9
*SlCLC02*	740	79,244.76	6.42	40.24	104.09	0.29	Cell membrane, mitochondrion	9
*SlCLC03*	784	86,467.38	8.82	37.85	113.37	0.32	Cell membrane	9
*SlCLC04*	763	84,897.49	7.55	33.23	113.42	0.35	Cell membrane	10
*SlCLC05*	788	86,502.39	8.68	35.13	105.81	0.37	Cell membrane	11
*SlCLC06*	756	80,599.42	6.77	48.41	95.24	0.07	Cell membrane, mitochondrion	9
*SlCLC07*	793	87,404.59	7.84	43.61	104.1	0.26	Cell membrane	12
*SlCLC08*	822	88,644.01	6.72	36.8	100.16	0.11	Mitochondrion	11
*SlCLC09*	845	92,385.04	8.79	35.83	108.58	0.36	Cell membrane	12

NAA, number of amino acids; MW, molecular weight; PI, theoretical pI; II, instability index; AI, aliphatic index; GAH, grand average of hydropathicity; PSL, prediction of subcellular localization; TMDs, number of putative transmembrane domains.

**Figure 2:**
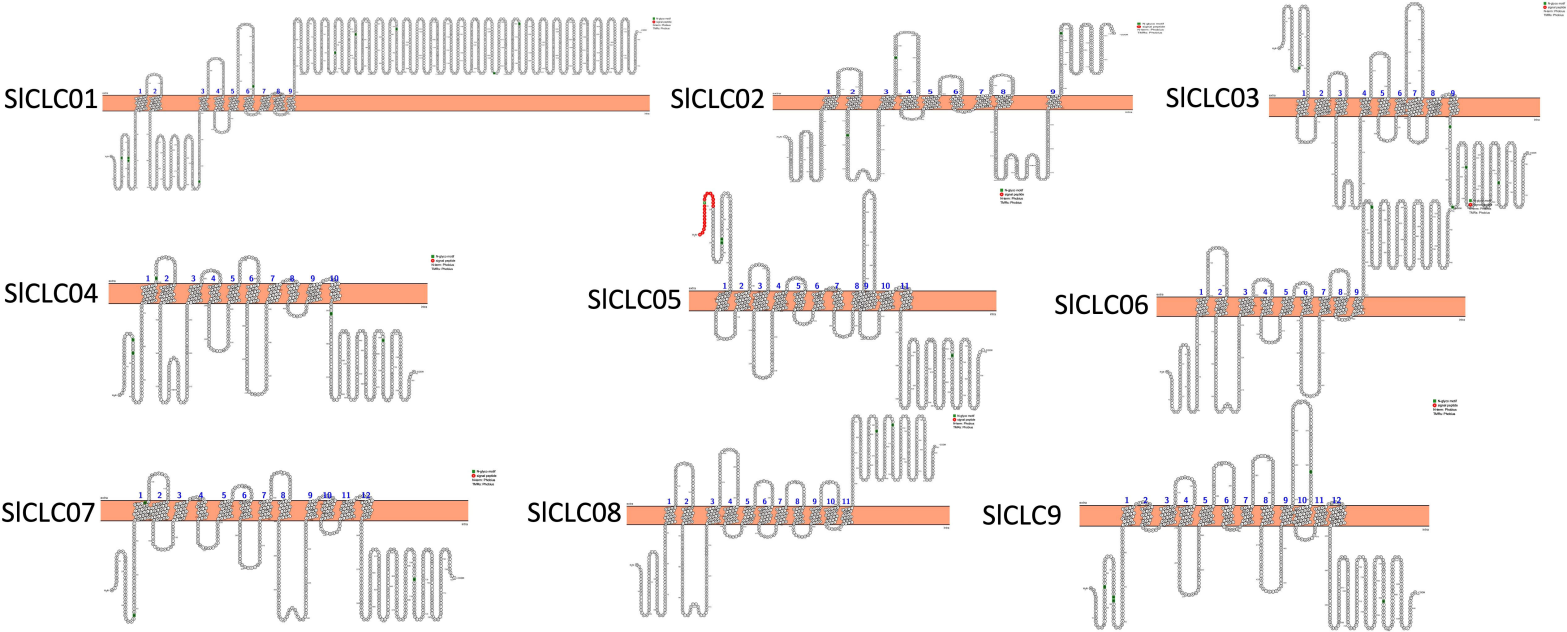
Predicted transmembrane domain of SlCLCs.

Furthermore, all the *Sl*CLC proteins were found on the plasma membrane and exhibited 9 to 12 transmembrane domains (**Table 1**; **Figure 2**). It is noteworthy that *Sl*CLC01 and *Sl*CLC08 were localized in the chloroplast and mitochondrion, respectively. *Sl*CLC02 and *Sl*CLC06 were found in both the cell membrane and mitochondrion, while the majority of *Sl*CLCs (*Sl*CLC03, *Sl*CLC04, *SlCLC05*, *Sl*CLC07, and *Sl*CLC09) were exclusively located in the cell membrane. The localization of proteins within subcellular compartments was fundamentally linked to their functional roles. Predicting the cellular locations of proteins is crucial for understanding gene functions. Further experimental confirmation is necessary to achieve more accurate subcellular localization.

Phylogenetic analysis plays a crucial role in examining gene function, the evolutionary relationships among species, and genetic diversity and variations. We constructed the phylogenetic tree using seven *CLC* proteins from *Arabidopsis thaliana*, 10 CLCs from *Glycine max*, 10 CLCs from *Solanum tuberosum*, 33 CLCs from *Triticum aestivum* L., and nine CLCs from *Solanum lycopersicum* L. to evaluate the evolutionary relationship. A phylogenetic tree was constructed based on 69 CLC proteins from *Arabidopsis*, soybean, wheat, potato, and tomato. All CLC proteins were classified into two groups (**Figure 1**). Each assembly covered members from diverse species, implying a high conservation among the CLC family. Group I included three SlCLC members (*Sl*CLC02, *Sl*CLC06, and *Sl*CLC08), and the other six *Sl*CLCs fell into Group II. Notably, *Sl*CLCs genes were found to have significant sequence similarity to StCLCs, except for *Sl*CLC07 which was similar to *At*CLC-D. These findings specify that the well-maintained sequences of these genes share a comparable evolutionary relationship in potato.

### 3.2 Conserved motifs, domains, and models of SlCLC proteins

**Figure 3:**
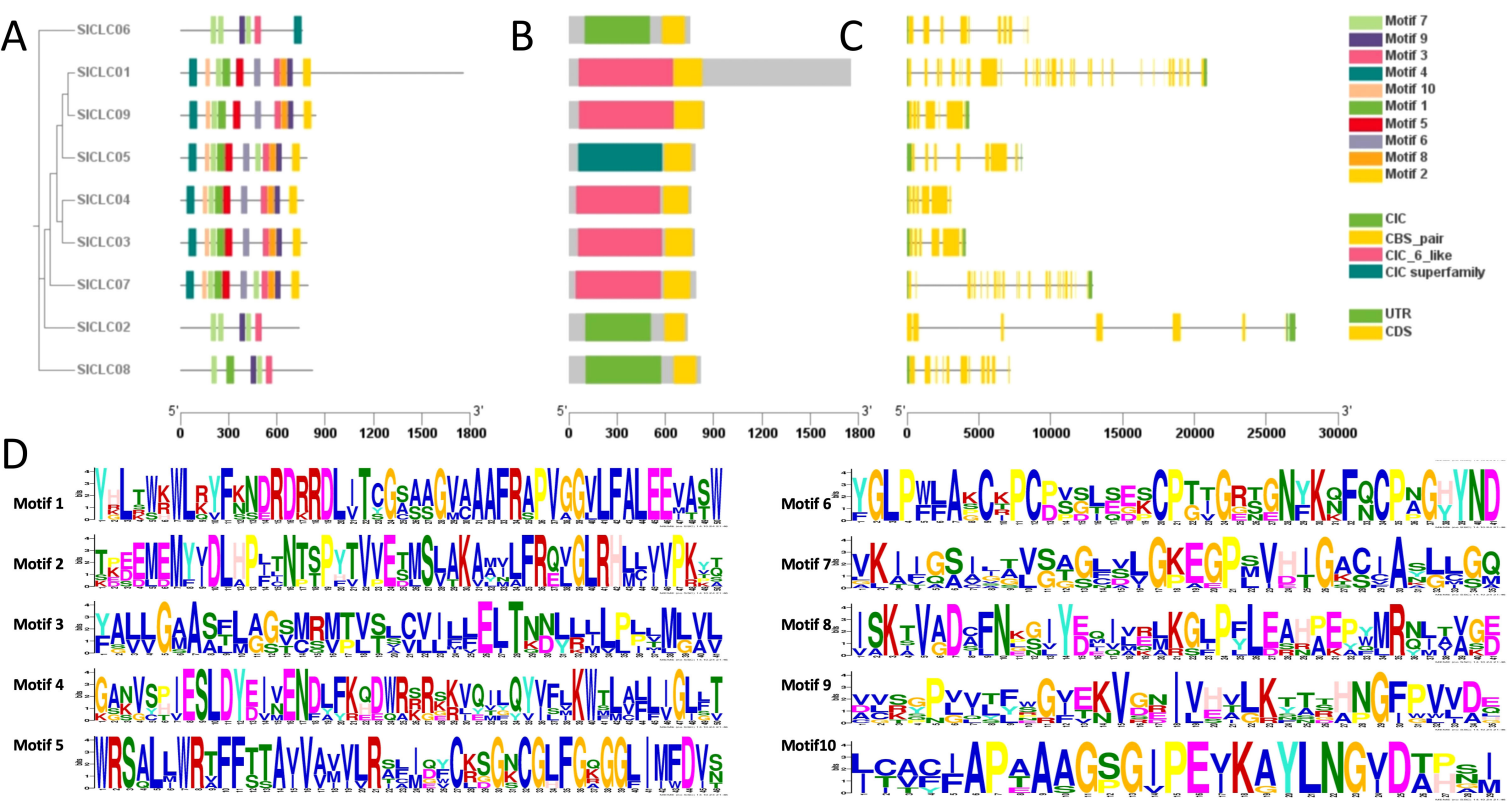
Conserved motifs **(A)** and domains **(B)** in SlCLC proteins and exon-intron structure **(C)** and amino acid composition of each motif **(D)**. UTR and CDS represent untranslated regions and coding sequences, respectively.

According to the protein structure analysis, nine SlCLC proteins were investigated, and a total of 10 conserved motifs were identified. Notably, significant differences were observed among the two different groups (**Figure 3A**). In fact, *Sl*CLC01, *Sl*CLC3, *Sl*CLC4, *Sl*CLC05, *Sl*CLC07, and *Sl*CLC09 from Group II contained all 10 motifs, but *SlCLC02*, *SlCLC06*, and *SlCLC08* particularly had motifs 3, 7, and 9, indicating a distinct evolutionary process and physiological function. All the SlCLC proteins contained one domain named CLC and CBS_pair, which is the typical domain of the CLC family (**Figure 3B**). The conserved amino acid residues consisted of motif 10 (GxGxPE), motif 7 (GKxGPxxH), and motif 1 (PxGxLF), including motif 3 (GxAxELT) and motif 9 (VxIxKxG) in particular (**Figure 3D**).

### 3.3 Exon-intron structure and duplication in the SlCLC gene family

**Figure 4:**
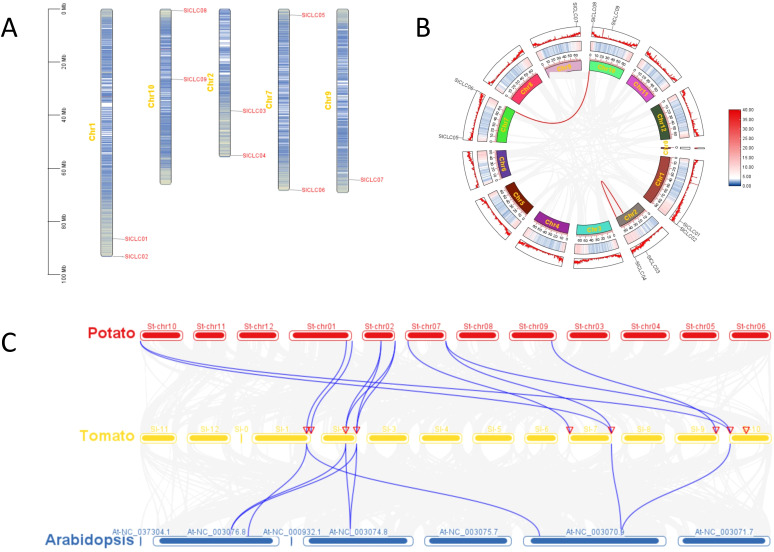
Schematic representations of the chromosomal location, synteny analyses, and segmental duplication relationships of *Sl*CLCs in tomato species. **(A)** chromosome location; **(B)** synteny analyses; **(C)** segmental duplication relationships.

A comparison of gene structures within the tomato species SlCLC gene family revealed significant variations in the number of introns, as shown in **Figure 3C**. When investigating the evolutionary mechanisms of the SlCLC gene family, our results show that the distribution of the nine genes was not uniform across all the chromosomes in tomato. *SlCLC01* and *SlCLC02* were found on chromosome 1, while *SlCLC08* and *SlCLC09* were distributed on chromosome 10. The genes *SlCLC03* and *SlCLC04* were located on chromosome 2, *SlCLC05* and *SlCLC06* were distributed on chromosome 7, and *SlCLC07* was located on chromosome 9 (**Figure 4A**).

To better understand the origin and functional divergence of the SlCLC gene family, a comparative collinearity analysis was conducted for CLC genes in tomato (**Figure 4B**). Within the tomato genome, two pairs of paralogous genes, *SlCLC06*/*SlCLC08* and *SlCLC03*/*SlCLC04*, were detected, and *SlCLC03* and *SlCLC04* were on the same chromosome (**Figure 4B**), signifying a segmented duplication role in the expansion of the *Sl*CLCs gene family. To further reveal the evolution of the CLC family, a comparative syntenic analysis was carried out on tomato, potato, and *Arabidopsis* (**Figure 4C**). A total of 12 and 8 collinear blocks were detected between tomato (eight genes) and potato (eight genes) and between tomato (five genes) and *Arabidopsis* (five genes), respectively (**Figure 4C**). Eight SlCLC genes were collinear with seven StCLCs of potato through the eight SlCLC genes, suggesting a close evolutionary relationship between these two species. All SlCLC genes were distinctly collinear with the CLCs, suggesting a high degree of homology and conservation of CLC genes across three species and a close evolutionary relationship among the other two species.

### 3.4 3D model predictions and cis-acting elements of *Sl*CLCs

**Figure 5:**
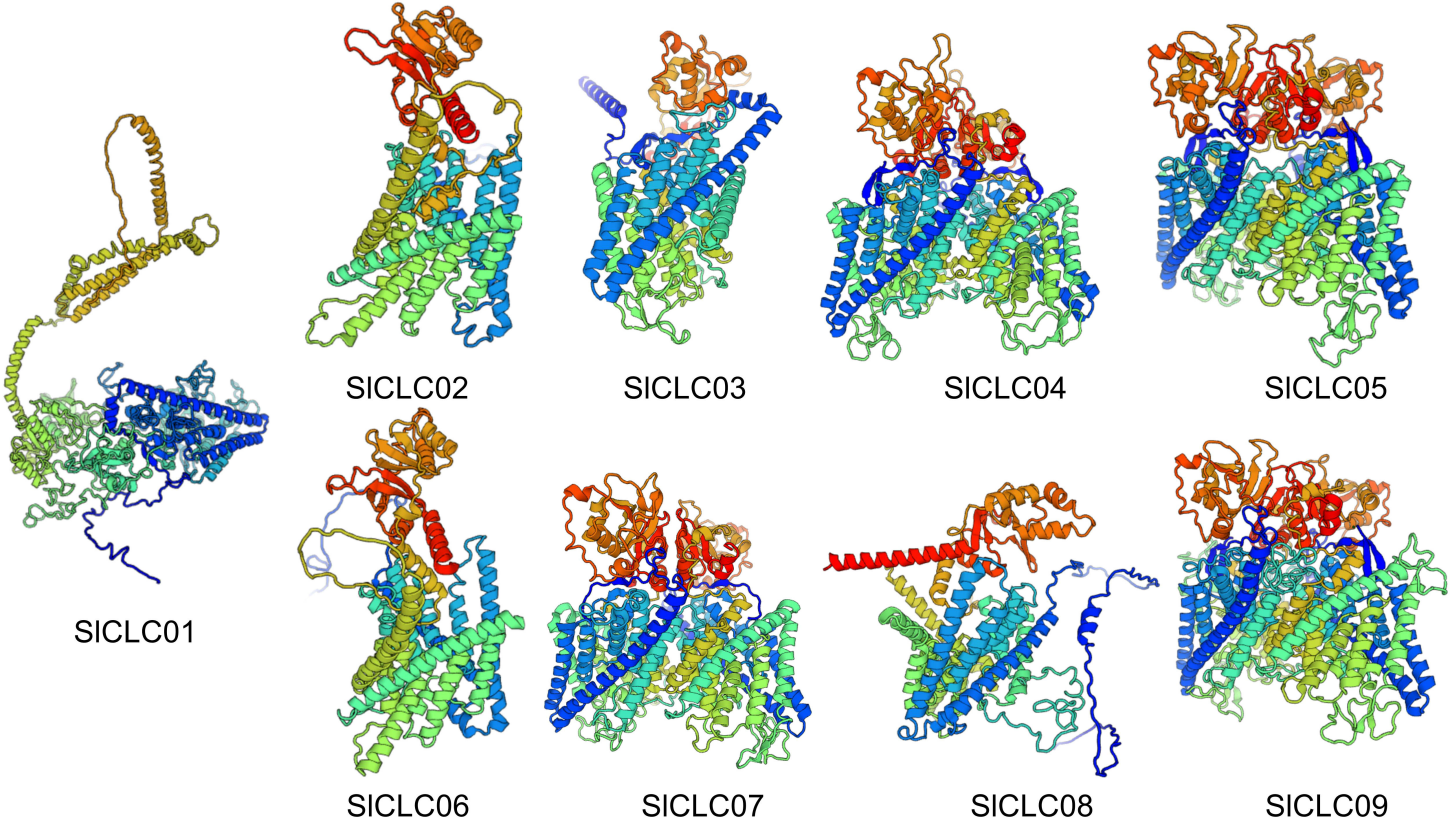
Predicted 3D structure of nine genes in the *Sl*CLCs family using a Swiss model.

**Figure 6:**
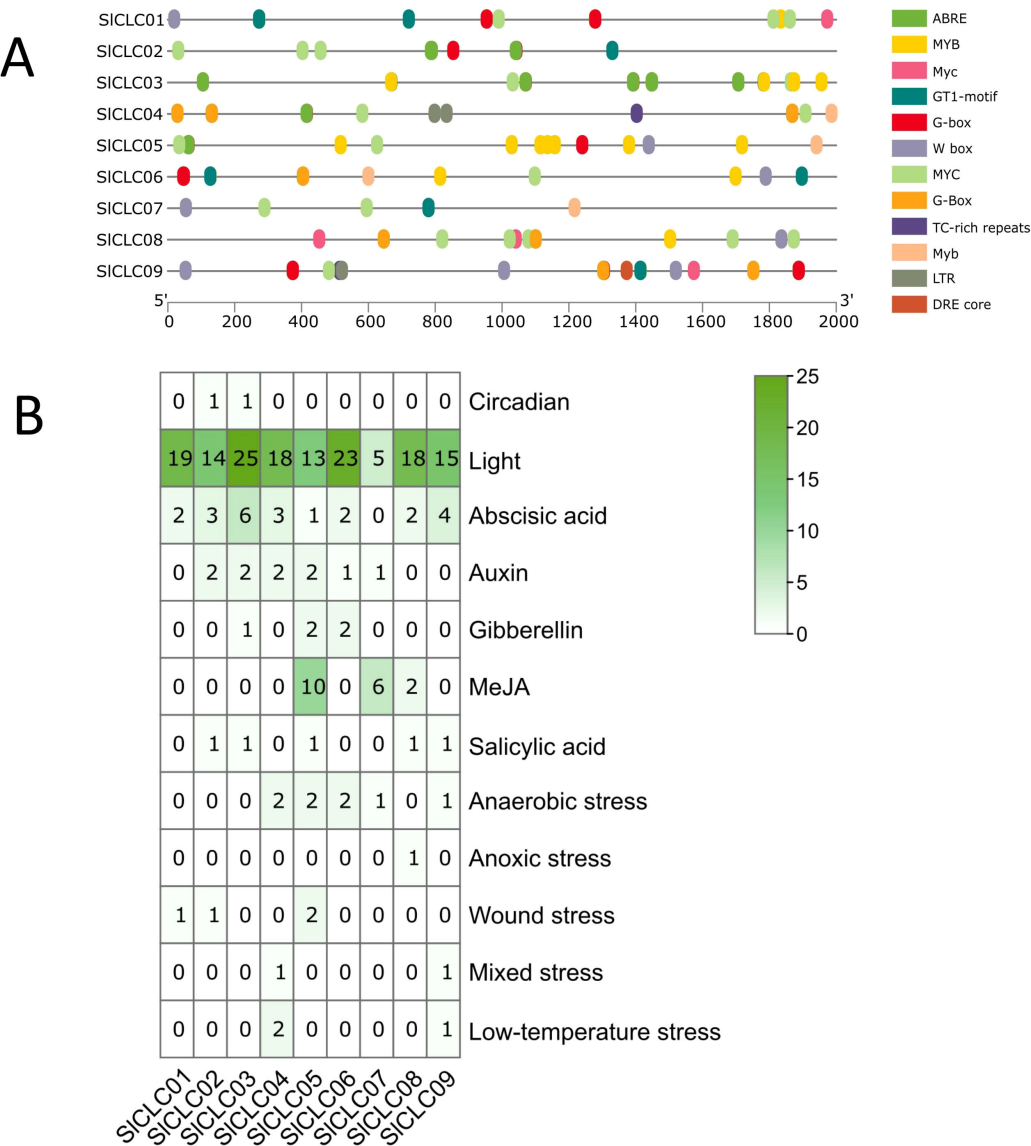
Identification of the cis-acting element in the promoter of SlCLC genes.

The three-dimensional structures of these SlCLC proteins were predicted using homology modeling. Noteworthy changes were detected in the three-dimensional architectures of the four proteins, as shown in **Figure 5**. The three-dimensional structures of these proteins serve as the basis for understanding their biological functions. In *Sl*CLC04, *Sl*CLC05, *Sl*CLC07, and *Sl*CLC09, distinct patterns of coiling and folding structures were evident, whereas *Sl*CLC02, *Sl*CLC03, *Sl*CLC08, and *Sl*CLC06 exhibited a relatively lower proportion of coiled regions. There were still significant differences in the 3D model protein predictions between *Sl*CLC01 before and after being restricted (**Supplementary Figure S2**). To better understand the transcriptional regulation and potential function of the SlCLC genes, the cis-regulatory elements in the promoter were predicted (**Supplementary Table S4**). In total, 36 functional cis-elements were obtained, and the top three were Box 4, G-box, and abscisic acid responsiveness element (ABRE). Their functions were part of a conserved DNA module, a cis-acting regulatory element involved in light responsiveness, and abscisic acid responsiveness, respectively. Hormone responsiveness and stress-related transcription factors were found in all nine *Sl*CLCs. MYC promoter elements were found in all nine *Sl*CLCs. MYB was found in eight *Sl*CLCs, except *SlCLC02*. Many hormone-related elements were found in the promoter regions of these *Sl*CLC*s* (**Figure 6A**, **Supplementary Table S4**). Among them, ABRE was identified in four *Sl*CLCs, a low temperature responsive (LTR) element was found in two *Sl*CLCs, and W box (a specific DNA sequence element, with the core sequence TTGACC/T) was found in six *Sl*CLCs, except *SlCLC02*, *SlClC03*, and *SlCLC04*. Our study uncovered multiple cis-regulatory elements that show a critical position in light, abiotic stress, and hormone responsiveness, as illustrated in **Figure 6B**.

This involved examining the 2,000 upstream base pairs of the CLC genes. These elements included circadian, light, ABA, auxin, gibberellin, methyl jasmonate (MeJA), and salicylic acid, and anaerobic, anoxic, wound, mixed, and low-temperature stresses. In fact, a salicylic acid responsiveness element was identified in *SlCLC02* and *SlCLC03*, and mixed and low-temperature responsive elements were identified in *SlCLC04*. In addition, three genes, *SlCLC05*, *SlCLC07*, and *SlCLC08*, were found to contain elements that are essential for gibberellin and MeJA hormone responsiveness. Anoxic stress elements were found in *SlCLC08*, especially. Stress-related elements were found in *SlCLC09*, such as mixed stress and low temperature. The findings indicate that SlCLC genes have a significant role in the stress responsiveness of tomato in the signaling networks that govern different developmental processes and also in response to biotic and abiotic stresses.

### 3.5 *Sl*CLC interaction networks

**Figure 7:**
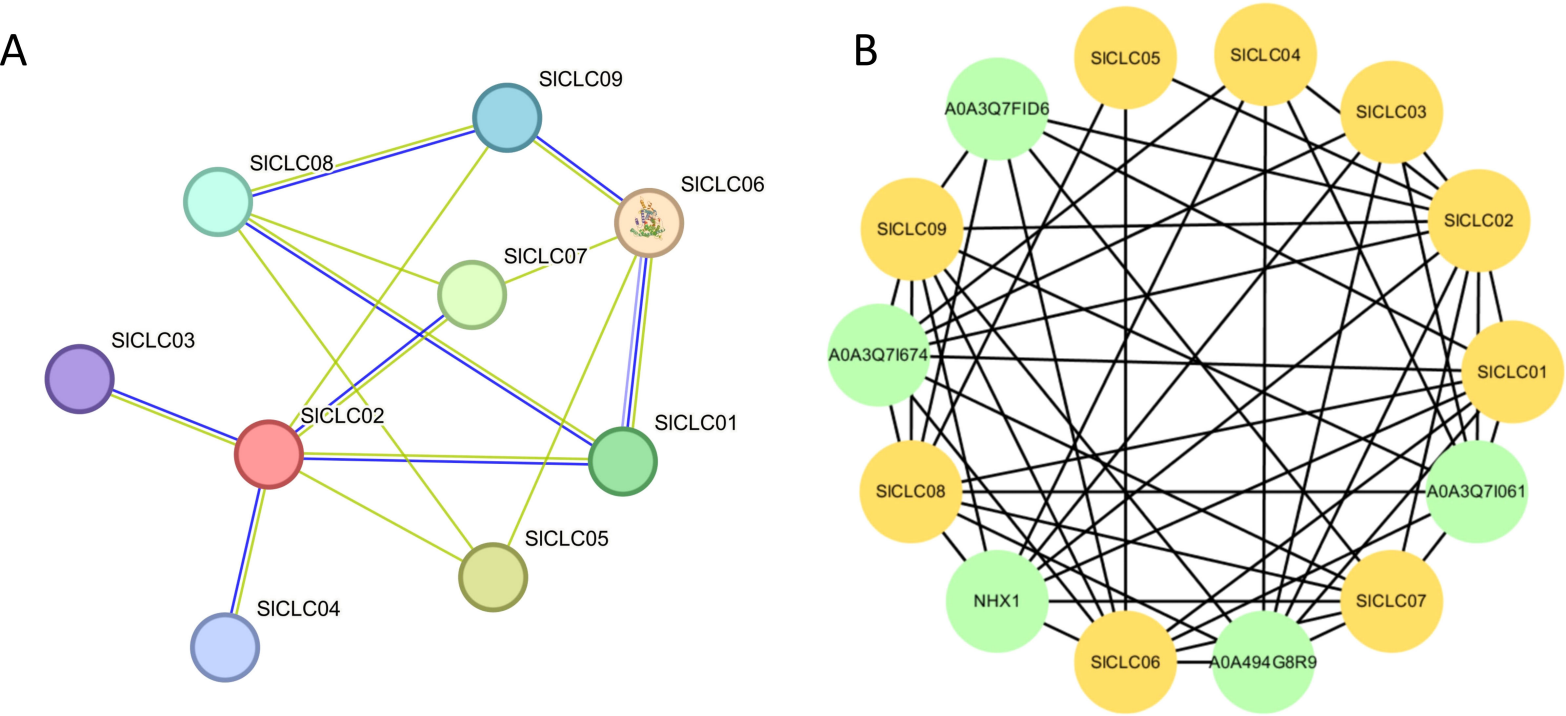
Modified protein–protein interaction (PPI) network based on SlCLC proteins. The PPI network shows the interaction relationships between CLC proteins. The two circles connected by the gray line represent the interaction between the proteins.

Protein-protein interactions (PPIs) were projected using the STRING tool. Nodes with higher connectivity were considered more critical for the stability of the entire network and we utilized Cytoscape software to refine it. *Sl*CLC02 exhibited higher connectivity with *Sl*CLC01, *Sl*CLC03, *Sl*CLC04, *Sl*CLC05, *Sl*CLC07, and *Sl*CLC09, excepting *Sl*CLC06 and *Sl*CLC08*. Sl*CLC08 interacted with *Sl*CLC01, *Sl*CLC05, *Sl*CLC07 and *Sl*CLC09, *Sl*CLC03, and *Sl*CLC04 (**Figure 7A**). In tomato, *Sl*CLC01, *Sl*CLC02, *Sl*CLC06, *Sl*CLC07, *Sl*CLC08, and *Sl*CLC09 were likely to be regulated by A0A3Q7FID6, which belongs to transmembrane transport, demonstrating transmembrane transporter activity. Specifically, *SlCLC07* was also connected with A0A3171674, A0A494G8R9, and A0A3Q71061. The functions of A0A3171674, A0A494G8R9, and A0A3Q71061 were ion binding, metal ion binding, transition metal ion binding, lipid transporter activity, and intramembrane lipid transporter activity. It is worth noting that *NHX1* annotation was sodium and potassium-proton antiporter activity, anion antiporter activity, and active ion transmembrane transporter activity (**Supplementary Table S5**; **Figure 7B**).

### 3.6 *SI*CLC expression patterns in a range of tissue types and prediction of miRNA targeting

**Figure 8:**
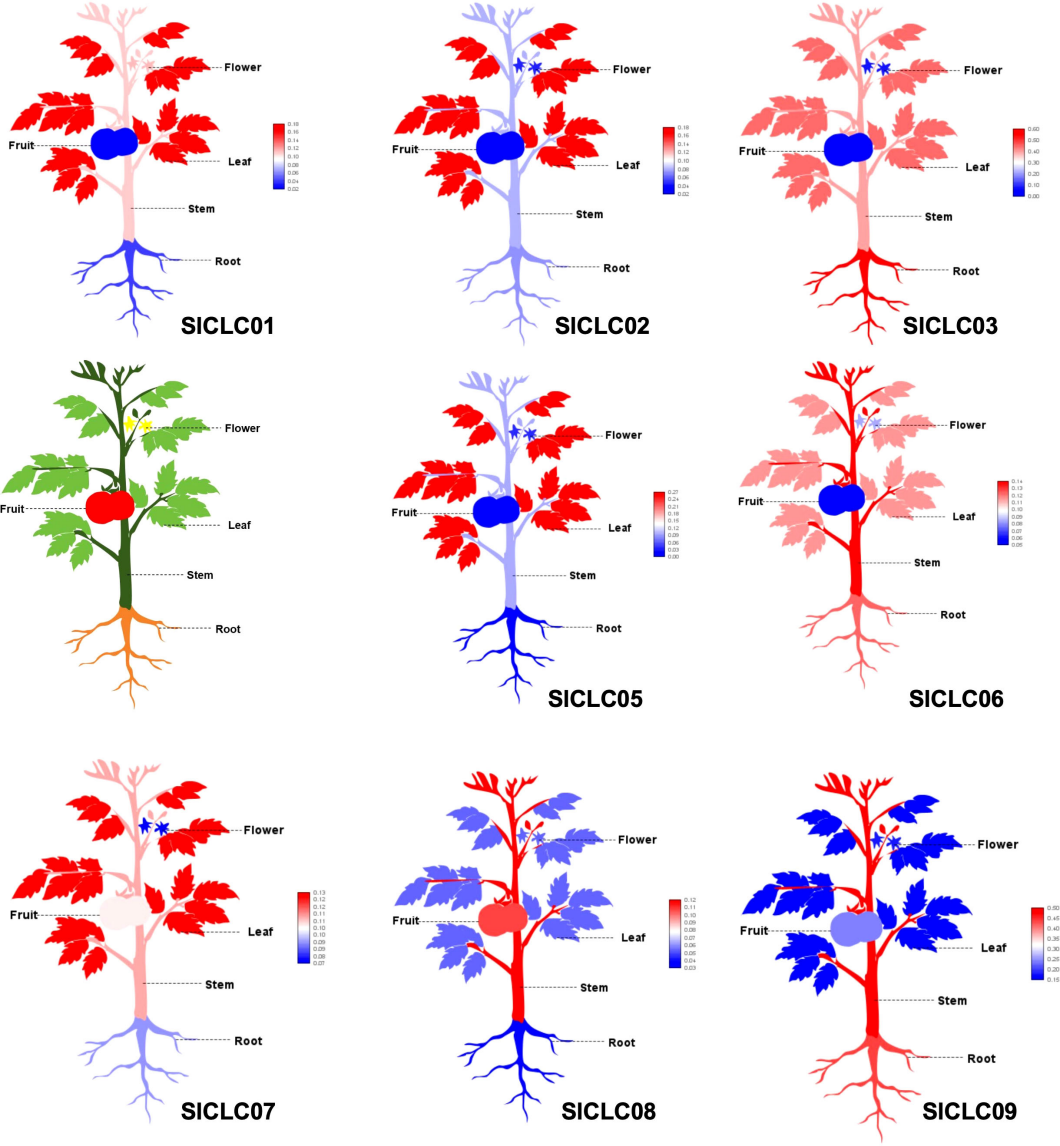
SlCLC gene expression patterns were evaluated in a range of tissue types. In total, 20 samples from three key developmental stages were collected for metabolic profiling and RNA-seq.

To determine the expression profiles of *Sl*CLCs genes in *Solanum lycopersicum*, a systematic analysis was performed across a range of plant tissues. Due to the lack of data for *SlCLC04* in the corresponding database, the data analysis was conducted excluding *SlCLC04*, and the findings showed unique transcription levels for each of the eight genes (**Figure 8**). *SlCLC01*, *SlCLC02*, *SlCLC05*, and *SlCLC07* showed significant expression in leaves. *SlCLC06*, *SlCLC08*, and *SlCLC09* displayed high expression levels in stems. It is noteworthy that the transcript levels of *SlCLC03* and *SlCLC09* were generally higher in roots than other members of *Sl*CLC across all plant tissues examined. Based on these findings, we can better understand how various organs in tomato plants regulate chloride ions via the SlCLC gene family. The prediction of miRNA targeting in whole-genome analysis is essential for unraveling the intricacies of gene regulation and can have significant implications for understanding developmental processes and responses to environmental stimuli. The prediction of miRNA targeting is shown in **Table 2**. The miRNAs sly-miR390a-5p, sly-miR390b-5p, sly-miR395a, sly-miR395b, and sly-miR482e-3p target the gene Solyc01T003527.1 (*SlCLC01*), and sly-miR5303 targets the gene Solyc10T001041.2 (*SlCLC09*), both of which are involved in inhibiting translation. Furthermore, other miRNAs are involved in the inhibition of cleavage.

**Table 2:** Prediction of miRNAs targeting the *SlCLC* genes in tomato.

miRNA_Acc.	Target_Acc.	Target site	miRNA_aligned_fragment	Alignment	Target_aligned_fragment	Inhibition
sly-miR167b-5p	Solyc01T004399.1	1086-1107	UAAAGCUGCCAGCAUGAUCUGG	**.::.:.:::::.:::::::**	UCUGGUUGGGCUGGUAGCUUUA	Cleavage
sly-miR9471a-3p	Solyc10T000071.2	1190-1210	UUGGCUGAGUGAGCAUCACGG	**.:::.::.:.::::.::.:.**	GUGUGGUGUUUACUCGGCUAG	Cleavage
sly-miR9471b-3p	Solyc10T000071.2	1190-1210	UUGGCUGAGUGAGCAUCACUG	**:::.::.:.::::.::.:.**	GUGUGGUGUUUACUCGGCUAG	Cleavage
sly-miR167a	Solyc01T004399.1	1087-1107	UGAAGCUGCCAGCAUGAUCUA	**:.:.:::::.:::::.:**	CUGGUUGGGCUGGUAGCUUUA	Cleavage
sly-miR395a	Solyc10T000071.2	1215-1236	CUGAAGUGUUUGGGGGAACUCC	**.::::.:.::.::.:::::**	CUGGUUCUCUAAAGCAUUUCAG	Cleavage
sly-miR395b	Solyc10T000071.2	1215-1236	CUGAAGUGUUUGGGGGAACUCC	**.::::.:.::.::.:::::**	CUGGUUCUCUAAAGCAUUUCAG	Cleavage
sly-miR390a-5p	Solyc10T001041.2	1645-1665	AAGCUCAGGAGGGAUAGCACC	**::::::.:::.:::.::**	AUUGCUAUUCCUUCUGGACUC	Cleavage
sly-miR390a-5p	Solyc01T003527.1	1633-1653	AAGCUCAGGAGGGAUAGCACC	**::::::.::.:::.:::**	AUUGCUAUUCCCUCUGGGCUG	Translation
sly-miR9476-3p	Solyc07T000272.1	2101-2121	AAAAAGAUGCAGGACUAGACC	**::::.:::::::::::**	UUUAUAGAUCUGCAUCCUUUU	Cleavage
sly-miR166c-5p	Solyc02T001059.1	721-741	GGGAUGUUGUCUGGCUCGACA	**:::.::::.:.::::.:**	GAUCGGGACAGGCGAGAUCUC	Cleavage
sly-miR390b-5p	Solyc01T003527.1	1633-1653	AAGCUCAGGAGGGAUAGCGCC	**.:::::.::.:::.:::**	AUUGCUAUUCCCUCUGGGCUG	Translation
sly-miR390b-5p	Solyc10T001041.2	1645-1665	AAGCUCAGGAGGGAUAGCGCC	**.:::::.:::.:::.::**	AUUGCUAUUCCUUCUGGACUC	Cleavage
sly-miR395a	Solyc01T003527.1	4018-4040	CUGAAGUGUU-UGGGGGAACUCC	**::::::::::::::::.:**	UAUGUUCCCCCACAACACUUUAU	Translation
sly-miR395b	Solyc01T003527.1	4018-4040	CUGAAGUGUU-UGGGGGAACUCC	**::::::::::::::::.:**	UAUGUUCCCCCACAACACUUUAU	Translation
sly-miR477-3p	Solyc07T002783.1	1700-1721	AGUUCUUGUAGGGUGAGACAAC	**::::::…::::::::**	AAGGUCUCAUUUUAGAAGAUCU	Cleavage
sly-miR9469-3p	Solyc01T003527.1	4619-4639	AUUCGGUCUUCUUAUGUGGAC	**:.::.:::::::.::.:**	CUUUAGCUGAGAAGACUGAGU	Cleavage
sly-miR9476-3p	Solyc09T002320.2	1519-1540	AAAAAGAUGCAGGA-CUAGACC	**:::::::::::::::::**	CUUCUAGGUGCUGCAUCUUUUC	Cleavage
sly-miR156d-5p	Solyc02T002924.1	383-402	UGACAGAAGAGAGUGAGCAC	**::::::::.:::.::::**	GUGCUAACUUUCUGUUGACA	Cleavage
sly-miR156e-3p	Solyc01T004399.1	1676-1696	GCUUACUCUCUAUCUGUCACC	**:::::::::::::::**	CUUCACAAAAAGAGAGUAACC	Cleavage
sly-miR156e-5p	Solyc07T000272.1	1378-1396	UGAUAGAAGAGAGUGAGCAC	**:::::.:.:::::::::**	AUGCUGAUUUUCUU-UAUCA	Cleavage
sly-miR164a-5p	Solyc02T001059.1	637-657	UGGAGAAGCAGGGCACGUGCA	**::.::::.::::::.:**	GGCGCUUGCUUUGCUUCCUUA	Cleavage
sly-miR164a-5p	Solyc02T002924.1	601-621	UGGAGAAGCAGGGCACGUGCA	**::::::.:::::::.:**	GGCAGUUGCAUUGCUUCUUUA	Cleavage
sly-miR164b-5p	Solyc02T001059.1	637-657	UGGAGAAGCAGGGCACGUGCA	**::.::::.::::::.:**	GGCGCUUGCUUUGCUUCCUUA	Cleavage
sly-miR164b-5p	Solyc02T002924.1	601-621	UGGAGAAGCAGGGCACGUGCA	**::::::.:::::::.:**	GGCAGUUGCAUUGCUUCUUUA	Cleavage
sly-miR482d-3p	Solyc02T001059.1	210-231	UUUCCUAUUCCACCCAUGCCAA	**:::::::::.::::.:**	CAAGCAUGACUGGAGAAGGAGA	Cleavage
sly-miR482e-3p	Solyc01T003527.1	4973-4994	UCUUUCCUACUCCUCCCAUACC	**::::::::::::.::**	AUAAUGUGAAGCGUAGGAGGGA	Translation
sly-miR5302a	Solyc07T002783.1	968-988	AAACGAGGUUUGUUACUUUGG	**…::::.::.:.::::**	GUGGAGUAGUAAGUGUUGUUU	Cleavage
sly-miR5302a	Solyc02T002924.1	639-659	AAACGAGGUUUGUUACUUUGG	**::::::::::.::::**	AGACAGUUACAAACUUAGUUG	Cleavage
sly-miR5303	Solyc10T001041.2	717-737	UUUUUGAAGAGUUCGAGCAAC	**::::::.:::::::::**	AUGGCUAAAAUACUUCAAAAA	Translation
sly-miR6027-5p	Solyc10T001041.2	1859-1880	AUGGGUAGCACAAGGAUUAAUG	**:.::::::::::.:::**	UGGUGAUGCUUGUUCUCCUCAU	Cleavage

### 3.7 *Sl*CLC expression patterns under salt stress condition

**Figure 9:**
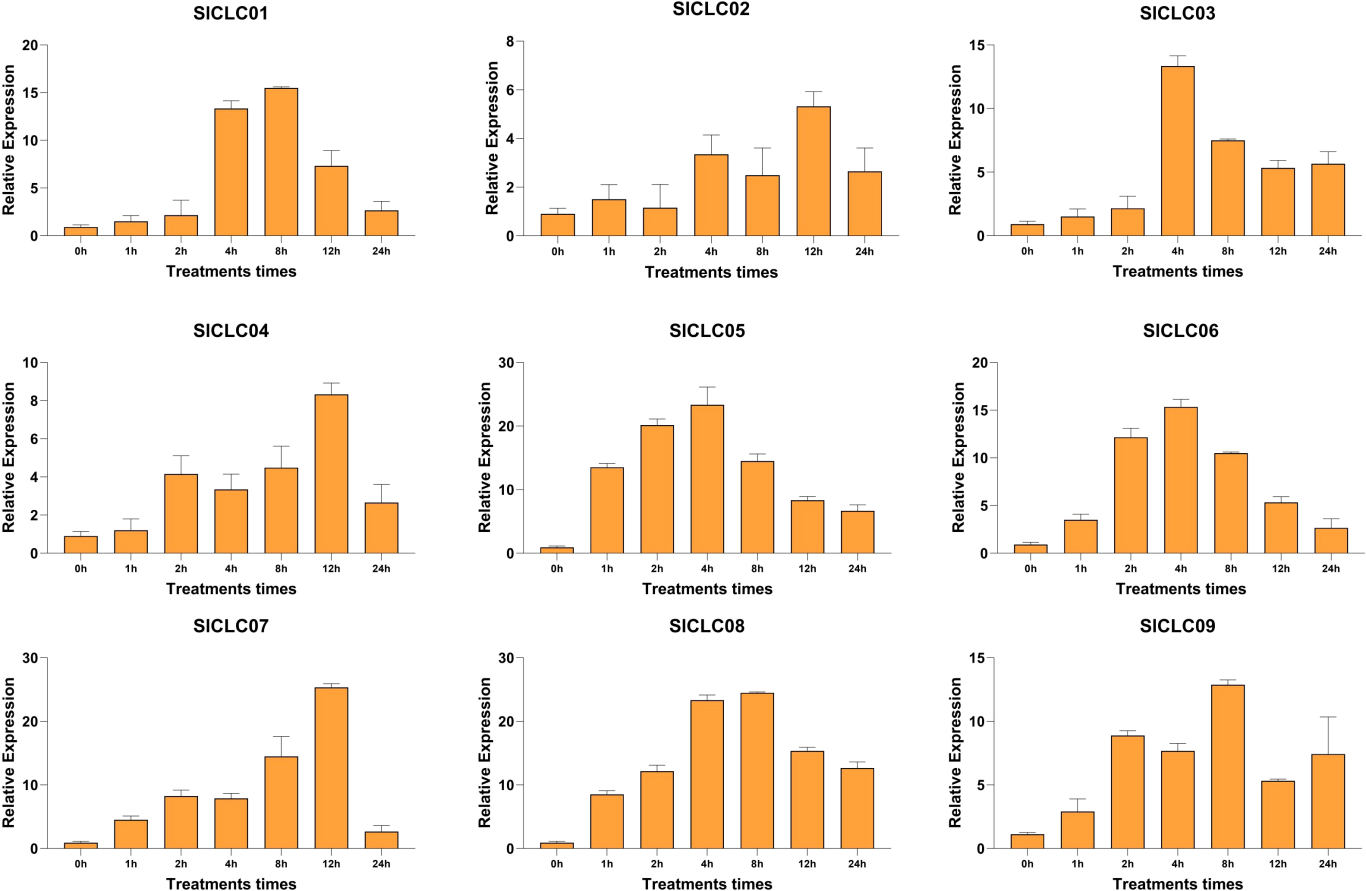
Expression patterns of *Sl*CLCs in tomato under NaCl stress.

To further investigate the response of *Sl*CLCs in tomato under salt stress, qRT-PCR was conducted to examine the expression patterns of these genes in plant roots, as demonstrated in **Figure 9**. After being treated with NaCl (100 mM), these *Sl*CLCs have different responses to environmental changes. Like *SlCLC01* and *SlCLC03*, the expression of *SlCLC02* in the roots was still low 4 hours after salt stress, but then *SlCLC02* expression was moderately upregulated compared with *SlCLC01* and *SlCLC03*. The response of *SlCLC05* was the most rapid, with its expression rapidly increasing during the first hour of salt stress treatment and maintaining a high level of expression for up to 8 hours. In contrast, the expression of *SlCLC07* increased slowly, reaching its peak at 12 hours. *SlCLC04* and *SlCLC07* also experienced a cliff-like drop after 12 hours, with a sharp decrease observed at the 24-hour mark. Other genes slowly declined after reaching their peak values. Similar to *SlCLC08*, *SlCLC06* exhibited a slow increase in expression over 24 hours, reaching its maximum at 4 hours, and then gradually decreased. The expression of *SlCLC09* exhibited fluctuation.

### 3.8 CLC gene expression pattern under Cd stress

**Figure 10:**
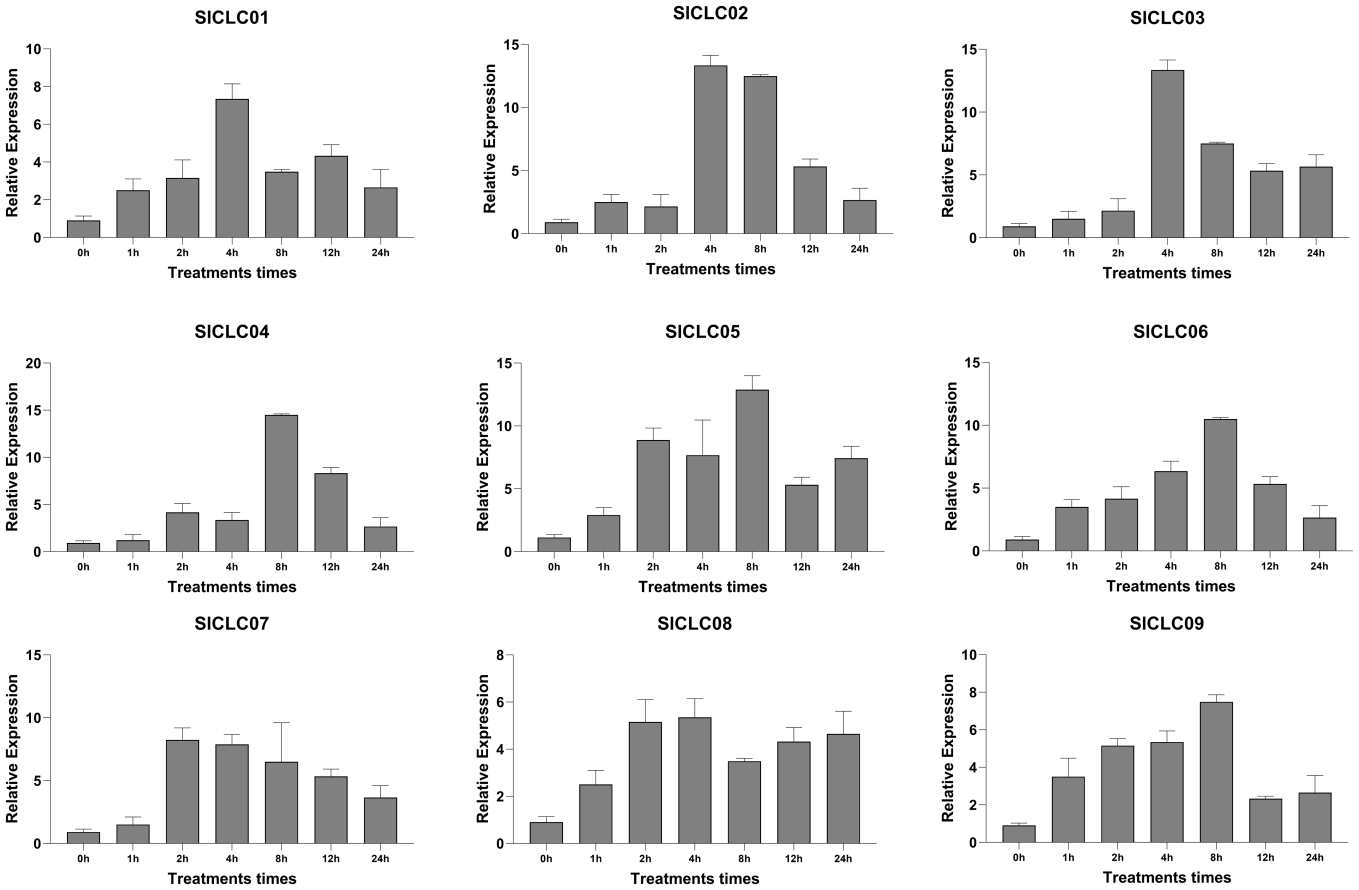
Expression patterns of *Sl*CLCs in tomato under Cd stress.

The expression of *SlCLC01* steadily increased during the cadmium stress treatment, reaching its maximal value at 4 hours and then gradually decreasing. At 4 hours, the expression of *SlCLC02* and *SlCLC03* significantly increased, a phenomenon that was also observed in other genes, including *SlCLC04* after 8 hours of stress treatment and *SlCLC05* and *SlCLC07* after 2 hours of stress treatment. The maximal values of *SlCLC06* and *SlCLC09* were achieved at 8 hours, respectively, even though they exhibited similar phenomena. Notably, *SlCLC02* and *SlCLC09* underwent an abrupt and substantial downregulation at 12 hours. After 2 hours of treatment, the *SlCLC08* gene exhibited a relatively stable upregulated expression, which persisted for 24 hours, as illustrated in **Figure 10**.

## 4 Discussion

Numerous CLCs have been found and functionally described as a result of their significant role in transporting anions across membranes and regulating ion homeostasis (Rajappa et al., 2024; Luan et al., 2025). Understanding the mechanisms of anion absorption and transport in plants is crucial for elucidating plant nutrition and stress responses. Although the association between CLC transporters and salt stress has been documented in certain species, such as *Arabidopsis thaliana* and *Glycine max* L (Liu et al., 2022; Rajappa et al., 2024), advancements in this area of research in other plants remain notably limited. Hence, this study undertook a comprehensive analysis of the CLC gene family within a tomato species using bioinformatics methodologies. Our objective was to provide novel insights into the function of the CLC gene family within the tomato species.

In this investigation, a total nine CLC family genes from genome of *Solanum lycopersicum*, designated as *SlCLC01* to *SlCLC09*, were discerned in other previously documented species, including *Arabidopsis thaliana* (7), *Glycine max* (10), *Solanum tuberosum* (10), and *Triticum aestivum* L. (33). Based on previously published investigations, it has been observed that numerous plant species possess up to 33 CLC genes. Our research findings align with previous studies, highlighting the conserved origin of the CLC gene family across diverse plant species. In concordance with previous studies that have characterized eukaryotic CLC proteins as containing two hydrophilic regulatory CBS domains, we observed that all nine tomato CLC proteins harbor these domains (**Figure 2**). During the structural analysis of the SlCLC protein, we found that the *Sl*CLCs contain a common conserved domain that was also found in other species. Our analysis also confirmed the presence of the three highly conserved amino acid motifs that are crucial for the formation of the anion conduction pathway (Nedelyaeva et al., 2020, 2023): the GxGxPE motif, which forms the selective filter in motif 10; the GKxGPxxH motif in motif 7; and the PxxGxLF motif in motif 1 (Bergsdorf et al., 2009; Wege et al., 2010). The selective filter in motif 10 is significant due to the presence of GSGxPE, which confers chloride specificity, and GPGxPE, which is responsible for nitrate specificity. Additionally, we postulate that the gating glutamate residue is located within motif 7, as in CLC proteins, the gating glutamate residue is situated within a conserved motif, GKxGPxxH. This motif is one of the three highly conserved regions in the CLC gene family, playing a crucial role in anion selectivity and transport function (Xing et al., 2020). The presence of the gating glutamate residue is a hallmark of CLC antiporters, which mediate active transport by coupling with energy consumption to move substrates against their electrochemical gradient (Zifarelli, 2022; Fortea et al., 2024). Although we have identified the genes containing motif 1 that potentially function in monomeric and homodimeric forms, the precise roles of these forms await further functional validation in future studies.

The cis-acting elements of CLC can provide more information about the regulation profiles of the SlCLC gene family. In this study, multiple cis-elements, which were identified from the promoter region of SlCLC family genes, directly respond to a variety of biotic and abiotic stresses, including anoxic stress, low-temperature stress, and wound stress. Nevertheless, there were ABRE, MeJA responsive elements, auxin-responsive elements, gibberellin-responsive elements, and salicylic acid-responsive elements. Our analysis showed that the MYC was prevalent in the promoters of all SlCLC genes, particularly in *SlCLC08*, which has five MYC binding sites. Given MYC’s role in jasmonate (JA)-mediated growth, development, and defense, it likely plays a significant part in the regulation of SlCLC genes under salt stress, especially in mitigating chloride toxicity (Fu et al., 2020). Additionally, the SlCLCs contained elements related to hormones, with MYB present in eight SlCLC genes (excluding *SlCLC02*) (**Figure 6**), therefore, CLC genes may have had some function related to plant growth and development during evolution. According to previous studies, a beneficial range of chloride (Cl^−^) applications can increase the ability of plants to tolerate drought stress (Franco-Navarro et al., 2021). Furthermore, CLCs have a relationship with the tolerance to salt and nitrate assimilation of plants (Li et al., 2024; Lundell and Biligetu, 2024). From the PPI results, we found a sodium proton antiporter NHX1 that interacted with *Sl*CLC03*, Sl*CLC06*, Sl*CLC07, and *Sl*CLC09 (**Supplementary Table S5**; **Figure 7B**), and it is worth noting that NHX1 also plays a crucial role in the developmental process and adaptation responses through phytohormonal signaling mechanisms (Ayadi et al., 2022). This also explains why these genes play a responsive role in plant hormone signaling as well. A recent study found that potatoes, closely related to tomatoes on the phylogenetic tree, were engineered to overexpress two genes from *Vitis vinifera*: *VvNHX*, a sodium/proton antiporter, and *VvCLC*, a chloride channel. The genetic modification led to enhanced plant growth, significantly improving tuber yield and quality. This indicates that the CLC and NHX genes may significantly influence tomato development (Ayadi et al., 2022).

To investigate the expression patterns of the SlCLC gene family in tomato (*Solanum lycopersicum*) under NaCl stress, this study conducted qRT-PCR analysis on nine genes of the SlCLC family. The results showed that the genes of the SlCLC family genes generally exhibited an expression pattern of initial upregulation followed by downregulation under NaCl stress. However, the expression patterns of SlCLC genes at various time points showed similarities with those in other species. In tobacco (*Nicotiana tabacum*), the expression levels of some NtCLC genes were significantly induced by salt stress (Zhang et al., 2018). In studies on soybean, where different chloride salts (MnCl_2_, KCl, and NaCl) were used for treatment, *GmCLC1* exhibited similar effects in alleviating the stress on yeast *GEF1* mutants caused by different chloride salts (Wei et al., 2016), however, the expression patterns of the genes cannot be solely attributed to chloride ions, as different effects have been observed in the expression patterns of CsCLCs in *Camellia sinensis* when utilizing KCl (Xing et al., 2020). Furthermore, although the expression of *SlCLC02* in the roots underwent moderate upregulation, it was still relatively low compared to other genes (**Figure 9**). This can be attributed to its predominant expression in leaves (**Figure 8**), a pattern shared by *SlCLC01*, *SlCLC05*, and *SlCLC07*. Interestingly, *SlCLC05* showed significant upregulation within the first hour of NaCl stress and was closely related to *AtCLCg* (**Figure 3**), and *AtCLCg* has been reported to participate in plant Cl^−^ homeostasis during NaCl stress (Nguyen et al., 2016). In other studies, the expression of *MhCLC-c1* was enhanced in response to NaCl stress, suggesting that these proteins are actively involved in Cl^−^ homeostasis and play a significant role in enhancing the plant’s overall salinity tolerance (Song et al., 2023). We confirmed that *SlCLC05* in tomato roots is highly sensitive and can rapidly respond to NaCl stress. Furthermore, we investigated *SlCLC03* and *SlCLC04*, which are closely related to the soybean *GmCLC-Aa* gene (**Figure 1**) (Wei et al., 2016). *GmCLC-Aa*, the first reported member, encodes a Cl^−^/H^+^ antiporter localized to the vacuolar membrane, contributing to Cl^−^ homeostasis under salt stress (Liu et al., 2022). Both *SlCLC03* and *SlCLC04* exhibited relatively high expression levels, with *SlCLC03* peaking rapidly at 4 hours before declining and dropping sharply after 12 hours. Given their phylogenetic relationship with *AtCLCa–AtCLCd* (De Angeli et al., 2006; von der Fecht-Bartenbach et al., 2010), *SlCLC03* and *SlCLC04* likely function as anion/proton antiporters and play a crucial role in rapidly responding to salt stress to maintain Cl^−^ homeostasis. *SlCLC06* and *SlCLC08*, which are phylogenetically close to *AtCLCf*, are speculated to play roles in chloride detoxification under salt stress (Rajappa et al., 2024). Furthermore, *AtCLCf* is also confirmed to be regulated by the *WRKY9* transcription factor, and increased intracellular NaCl levels can induce the translocation of *At*CLCf from the Golgi apparatus to the plasma membrane. However, the specific function of *SlCLC06* and *SlCLC08* still needs further experimental verification.

Currently, the expression patterns of the CLC gene family are highly variable and largely determined by the specific tissues sampled. The expression profiles of CLC genes are highly dependent on the sampling locations. For instance, the expression of TaCLC genes sampled from whole plants showed a downregulated expression pattern under transient salt stress (Mao et al., 2022). In contrast, the expression of SlCLC genes in plant roots initially increased and then decreased. Notably, even within the same plant, expression patterns can vary significantly among different tissues. Transcriptomic data from the TomExpress website suggest that this differential expression pattern may be attributed to the tissue-specific regulation of SlCLC genes (**Figure 8**), similar to the expression patterns observed for *GhCLC5/16* genes in cotton (Yang et al., 2023b). Additionally, comparative synteny analysis revealed that SlCLC09 does not share orthologous relationships with CLC genes from potato or *Arabidopsis thaliana* (**Figure 4C**). Collectively, these findings indicate that the SlCLC gene family has undergone functional differentiation during its evolutionary history.

Cadmium is mobilized through the phloem, allowing it to accumulate in any part of the plant (Zulfiqar et al., 2022). It leads to a reduction in biomass and yield due to its ability to induce membrane lipid peroxidation and competition for the Ca-calmodulin binding sites between Cd and Ca ions (Haider et al., 2021). Chloride ions can form CdCl^+^, which has less adsorption than Cd^2+^ because of the high exchange selectivity of the divalent ion (Saeki and Kunito, 2012). In our study, *SlCLC01*, *SlCLC02*, and *SlCLC03* exhibited gradual increases in expression, peaking at 4 hours before declining. *SlCLC04* and *SlCLC06* showed significant upregulation after 8 hours of stress treatment, while *SlCLC05* and *SlCLC07* displayed marked increases after only 2 hours. The earlier peak expression pattern of *SlCLC05* and *SlCLC07* shows their function in the early phases of the stress response and may be related to certain defensive mechanisms. The expression of the *SlCLC08* and *SlCLC09* genes increased slowly and resulted in a stably coordinated reaction that sequesters or transports cadmium away from sensitive cellular components, which is necessary for survival. The declining expression of these genes after a peak may indicate stress-related adaptation. Adaptation to cadmium stress depends on chloride channels, notably those carried by *CLC* genes, which control chloride ion flow and hence reduce cadmium toxicity (Fu et al., 2020). *SlCLC02* and *SlCLC09* downregulation after 12 hours may indicate a shift in the plant’s strategy for sustained stress control. These reactions indicate a sophisticated and adaptable regulatory network that allows plants to react to different degrees of stress over time.

## 5 Conclusion

**Figure 11:**
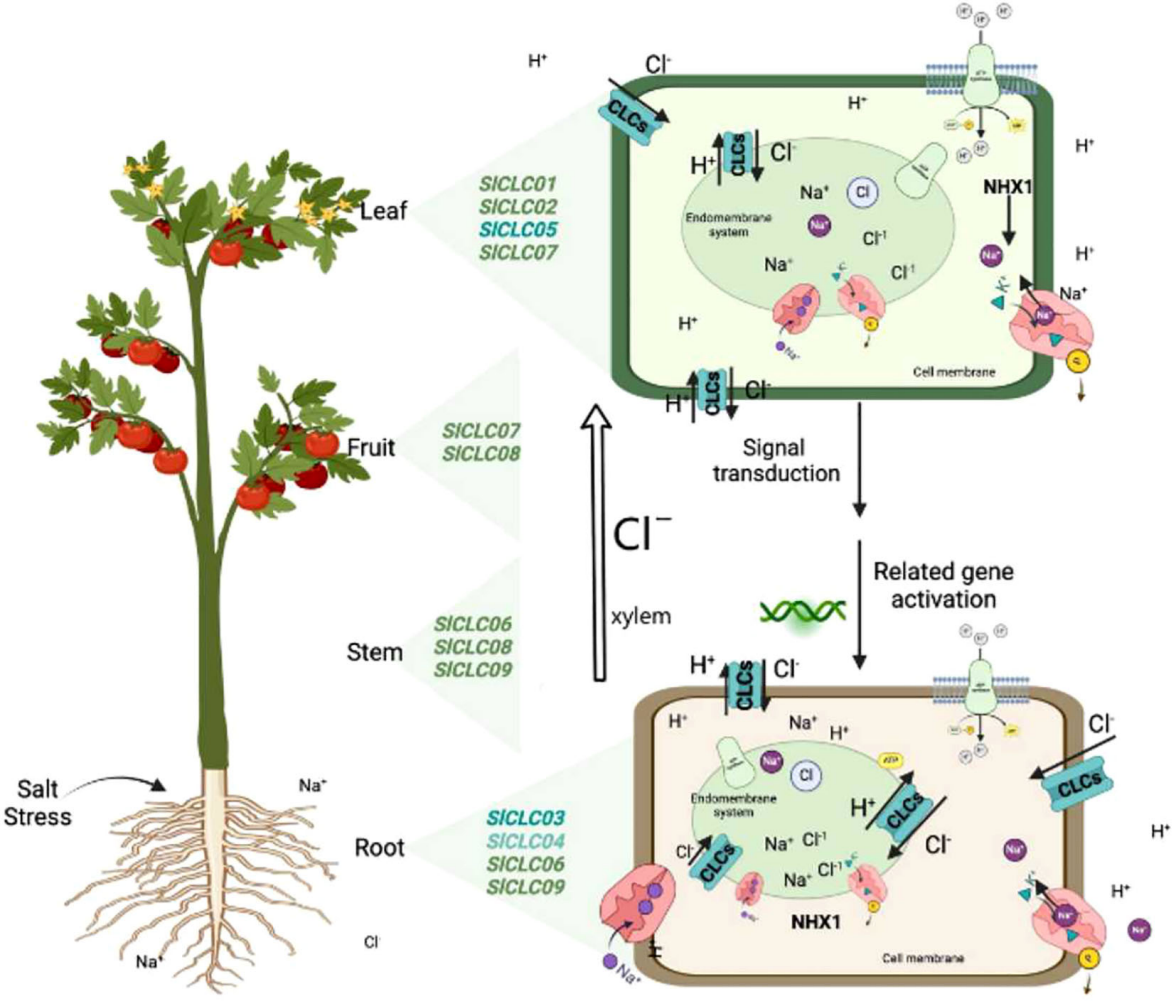
Diagram of SlCLC transport functions and expression locations in tomato. The left side shows the expression sites of SlCLCs based on expression profiles. The right side depicts the localization of SlCLCs in the membrane system and the possible cations involved in intracellular ion homeostasis.

In this study, we identified nine CLC family genes in tomato and analyzed their sequences and genetic structures. A qRT-PCR-based analysis showed the response patterns of the CLC genes under NaCl stress, thus providing genetic resources for studying the transportation and accumulation of Cl^−^ in tomato. The results indicate an intricate relationship between these genes and the plant’s capability to regulate chloride ion balance (**Figure 11**). The SlCLC gene’s promoter contains a variety of cis-acting elements, including light response, abscisic acid response, auxin response gibberellin, MeJA, salicylic acid, and anaerobic. The interactions between the proteins were strong, although *Sl*CLC02 had low sensitivity to NaCl stress but it plays an important role in coordinating function with other CLC genes. *Sl*CLC05 may have a physiological function in chloride homeostasis during NaCl stress due to its quick response to stress, and *Sl*CLC03 and *Sl*CLC04 play important roles in maintaining the balance of Cl^−^ by acting as anion/proton antiporters. Future studies must focus on supporting the computational predictions by means of lab-based experiments and more investigations into the functions performed by CLC genes in salinity tolerance. Such efforts might be very helpful in promoting the development of crop varieties with increased tolerance to environmental problems such as salinization.

## Data Availability

The original contributions presented in the study are included in the article/**Supplementary Material**. Further inquiries can be directed to the corresponding authors.
