# Supplementary material for: CLC gene family in Solanum lycopersicum: genome-wide identification, expression, and evolutionary analysis of tomato in response to salinity and Cd stress
Source: Front Plant Sci. 2025 Apr 29;16:1547723. doi: 10.3389/fpls.2025.1547723 (PMC12069385; doi:10.3389/fpls.2025.1547723)
Supplement: Supplementary file 1 [file DataSheet1.pdf]

FGENESH 2.6 Prediction of potential genes in Tomato genomic DNA
Seq name:  test sequence
Length of sequence: 20890
Number of predicted genes 2: in +chain 2, in -chain 0.
Number of predicted exons 29: in +chain 29, in -chain 0.
Positions of predicted genes and exons: Variant 1 from 1, Score:464.376855

CDSf   CDSi   CDSl   CDSo   PolA   TSS

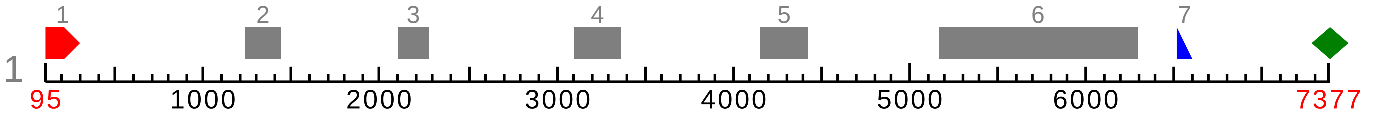


| | | | | | | | | | | | |
|---|---|---|---|---|---|---|---|---|---|---|---|
| 1 | + | 1 | CDSf | 95 | - | 291 | 14.56 | 95 | - | 289 | 195 |
| 1 | + | 2 | CDSi | 1228 | - | 1428 | 18.72 | 1229 | - | 1426 | 198 |
| 1 | + | 3 | CDSi | 2092 | - | 2269 | 27.15 | 2093 | - | 2269 | 177 |
| 1 | + | 4 | CDSi | 3093 | - | 3355 | 28.11 | 3093 | - | 3353 | 261 |
| 1 | + | 5 | CDSi | 4148 | - | 4417 | 29.47 | 4149 | - | 4415 | 267 |
| 1 | + | 6 | CDSi | 5158 | - | 6286 | 121.51 | 5159 | - | 6286 | 1128 |
| 1 | + | 7 | CDSl | 6508 | - | 6597 | 2.40 | 6508 | - | 6597 | 90 |
| 1 | + | | PolA | 7377 | | | 1.61 | | | | |

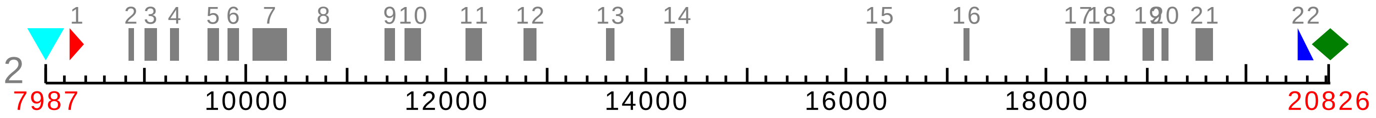


| | | | | | | | | | | | |
|---|---|---|---|---|---|---|---|---|---|---|---|
| 2 | + | | TSS | 7987 | | | -8.68 | | | | |
| 2 | + | 1 | CDSf | 8226 | - | 8369 | 18.07 | 8226 | - | 8369 | 144 |
| 2 | + | 2 | CDSi | 8813 | - | 8863 | 8.38 | 8813 | - | 8863 | 51 |
| 2 | + | 3 | CDSi | 8976 | - | 9096 | 8.08 | 8976 | - | 9095 | 120 |
| 2 | + | 4 | CDSi | 9230 | - | 9319 | 1.43 | 9232 | - | 9318 | 87 |
| 2 | + | 5 | CDSi | 9604 | - | 9716 | 11.88 | 9606 | - | 9716 | 111 |
| 2 | + | 6 | CDSi | 9802 | - | 9916 | 7.13 | 9802 | - | 9915 | 114 |
| 2 | + | 7 | CDSi | 10053 | - | 10395 | 35.33 | 10055 | - | 10393 | 339 |
| 2 | + | 8 | CDSi | 10689 | - | 10836 | 30.42 | 10690 | - | 10836 | 147 |
| 2 | + | 9 | CDSi | 11376 | - | 11477 | 3.87 | 11376 | - | 11477 | 102 |
| 2 | + | 10 | CDSi | 11577 | - | 11737 | 9.30 | 11577 | - | 11735 | 159 |
| 2 | + | 11 | CDSi | 12184 | - | 12346 | 0.31 | 12185 | - | 12346 | 162 |
| 2 | + | 12 | CDSi | 12765 | - | 12892 | 12.51 | 12765 | - | 12890 | 126 |
| 2 | + | 13 | CDSi | 13588 | - | 13672 | 2.50 | 13589 | - | 13672 | 84 |
| 2 | + | 14 | CDSi | 14231 | - | 14363 | 13.50 | 14231 | - | 14362 | 132 |
| 2 | + | 15 | CDSi | 16282 | - | 16358 | 6.06 | 16284 | - | 16358 | 75 |
| 2 | + | 16 | CDSi | 17161 | - | 17217 | 7.40 | 17161 | - | 17217 | 57 |
| 2 | + | 17 | CDSi | 18232 | - | 18379 | 6.03 | 18232 | - | 18378 | 147 |
| 2 | + | 18 | CDSi | 18463 | - | 18620 | 27.56 | 18465 | - | 18620 | 156 |
| 2 | + | 19 | CDSi | 18948 | - | 19061 | 25.83 | 18948 | - | 19061 | 114 |
| 2 | + | 20 | CDSi | 19138 | - | 19205 | 9.89 | 19138 | - | 19203 | 66 |
| 2 | + | 21 | CDSi | 19482 | - | 19654 | 17.94 | 19483 | - | 19653 | 171 |
| 2 | + | 22 | CDSl | 20500 | - | 20660 | 21.17 | 20502 | - | 20660 | 159 |
| 2 | + | | PolA | 20826 | | | -4.99 | | | | |

Predicted protein(s):
>FGENESH:[mRNA] 1 7 exon (s) 95 - 6597 2328 bp, chain +
ATGAGAAAGCAAGAAGATATAGAAAATGAAGGAGTTGGGGTAATGGTAATGGAAGATGGG
AAGGATTTAGAGAGGAATATTTCATCTGAGGGTGGTTTTAGAGAGCCATTGCTTAAATCA
AAGAGTAGAGTCAATAATACATCACAAATTGCTATTATTGGAGCCAATGTTTGCCCTATT
GAGAGTCTTGATTATGACATTGTTGAAAATGACCTATTCAAGCAAGATTGGAGATCAAGG
AAAAAGGTCCAGATATATCAATATATATTCCTTAAGTGGACACTCGTGCTTCTTATTGGA
TTGTTTACAGGACTTGTTGGTTTCTTTTTAAACATAGCAGTGGAAAATATTGCTGGCTTT
AAGCTTCTGCTTGCTAGTGATTTAATGCTCGAGGATAAGTATTTCCGTGCATTTGCTATT
TTTGCAGGTTGCAATTTGGGTCTTGCAACTTGTGCTGCCATCCTATGTGCATGTATTGCA
CCTGCAGCTGCAGGGTCAGGAATTCCTGAAGTTAAAGCATATTTGAATGGTGTGGATGCT
CATTCCATTTTAGCTCCTAGTACTTTATTTGTAAAGATAATTGGTTCTGTTTTGGGTGTT
TCTGCTGGATTTGTTGTTGGTAAGGAAGGACCCATGGTCCACACTGGCGCTTGCATAGCT
AACTTACTTGGGCAGGGCGGCTCCCGCAAATATCATCTGACGTGGAAGTGGCTGAGGTAT
TTTAAAAATGACCGTGACCGCAGAGATTTGATCACTTGTGGTGCTGCAGCTGGTGTTGCA
GCCGCTTTCCGTGCTCCAGTTGGTGGTGTTCTTTTTGCTCTCGAAGAAGTAGCCTCATGG
TGGCGAAGTGCTCTACTTTGGAGGACTTTCTTCACAACTGCTATAGTAGCTATGGTGCTC
AGATCTTGCATTCAATTCTGTCGGAGTGGGAACTGTGGACTATTTGGTCAAGGAGGTTTG
ATAATGTTTGATGTGAATTCAGGATTTCCTAATTATAACACTGTAGATGTACTGGCGGTA
TTGACAATTGGAGTTCTTGGAGGCCTTTTAGGAAGCCTTTATAATTATCTTGTGGACAAG
GTCCTGCGGACTTACAGCATCATTAATGAGAGAGGTCCTGCTTTCAAAATCATGCTCGTA
ATGACCATTTCGATCCTGACTTCTCTTTGTGCATATGGTCTTCCATGGTTTGCAAGTTGC
ACACCGTGCCCCGTAGGCTTGGAGGAAAAATGTCCTACTGTAGGTCGCTCTGGAAACTAT
AAGAATTTCCAGTGTCCGGCTGGGCATTACAATGATCTGGCCTCCCTGTTTATGAATACC
AATGATGACGCCATCCGCAATTTGTTTAGTGCAGAAAATTCAAGTGAATTCCACCTTTCT
ACACTTTTTGTCTTCTTCGCTGGGGTATACTGCCTTGGCATTATTACCTATGGAATTGCT
ATTCCCTCTGGGCTGTTCATTCCTGTCATACTTGCTGGAGCCTCATATGGACGTCTTGTT
GGAAGTGTCTTAGGTTCGGTCTCTAATCTTAATAATGGCCTGTTTGCGCTCCTTGGGGCT
GCCTCCTTCCTTGGTGGTACTATGAGGATGACAGTATCACTCTGTGTCATACTACTTGAG
CTCACCAATAATCTGCTAATGCTTCCGTTGGTGATGCTTGTTCTCCTTGTATCAAAAACT
GTGGCCGATAGTCTTAACAAGGGTATCTATGACCAGATTGTGCAAATGAAAGGCTTGCCT
TACTTGGAAGCACACGCCGAGCCTTACATGAGGCAATTGGCTGCAGGAGATGTTTGTTCT
GGGCCTTTAGTAACATTTTCAGGTGTTGAGAAGGTAGGAAACATAGTACACGCTTTGAAG
TTTACTCGACACAATGGGTTTCCCGTGGTTGATTTACCACCATTCTCAGACGCGCCAGAG
TTTTGTGGGCTTGTTTTAAGGTCACATTTAGTTGTTTTGCTCAAAGGAAAGACATTCACG
AAACAAAATGTACTGAGTGGCTCCAATACTCTGAAGAAGTTTCATGCATTTGATTTTGCG
AAGCCAGGATCAGGGAAGGGGCTTAAGTTTGAGGATTTGTCCTTCTCCCCCGAGGAGATG
GAAATGTATGTTGATCTCCATCCTATCACAAATACATCTCCATACACAGTAGTGGAAACC
ATGTCTCTGGCCAAAGCTGCAATTCTTTTCCGTGAACTCGGTCTCAGACATTTGTGTGTC
GTCCCAAAGACTACCAAGAGAAATCCAATAGTTGGAATCTTGACAAGGCATGACTTCATG
CCAGAGCATATAAAGGGACTGTATCCACATTTGGTCCATCACAAGTAA
>FGENESH: 1 7 exon (s) 95 - 6597 775 aa, chain +
MRKQEDIENEGVGVMVMEDGKDLERNISSEGGFREPLLKSKSRVNNTSQIAIIGANVCPI
ESLDYDIVENDLFKQDWRSRKKVQIYQYIFLKWTLVLLIGLFTGLVGFFLNIAVENIAGF
KLLLASDLMLEDKYFRAFAIFAGCNLGLATCAAILCACIAPAAAGSGIPEVKAYLNGVDA
HSILAPSTLFVKIIGSVLGVSAGFVVGKEGPMVHTGACIANLLGQGGSRKYHLTWKWLRY
FKNDRDRRDLITCGAAAGVAAAFRAPVGGVLFALEEVASWWRSALLWRTFFTTAIVAMVL
RSCIQFCRSGNCGLFGQGGLIMFDVNSGFPNYNTVDVLAVLTIGVLGGLLGSLYNYLVDK

VLRTYSIINERGPAFKIMLVMTISILTSLCAYGLPWFASCTPCPVGLEEKCPTVGRSGNY
KNFQCPAGHYNDLASLFMNTNDDAIRNLFSAENSSEFHLSTLFVFFAGVYCLGIITYGIA
IPSGLFIPVILAGASYGRLVGSVLGSVSNLNNGLFALLGAASFLGGTMRMTVSLCVILLE
LTNNLLMLPLVMLVLLVSKTVADSLNKGIYDQIVQMKGLPYLEAHAEPYMRQLAAGDVCS
GPLVTFSGVEKVGNIVHALKFTRHNGFPVVDLPPFSDAPEFCGLVLRSHLVVLLKGKTFT
KQNVLSGSNTLKKFHAFDFAKPGSGKGLKFEDLSFSPEEMEMYVDLHPITNTSPYTVVET
MSLAKAAILFRELGLRHLCVVPKTTKRNPIVGILTRHDFMPEHIKGLYPHLVHHK
>FGENESH:[mRNA] 2 22 exon (s) 8226 - 20660 2853 bp, chain +
ATGGAAGCGATCGAGGAATTGGAGCAGCTCGGCGATGCGATGAGGCAAGCCGCTGCGTTG
TTAGCCGATGAGGATGTCAATGAAGCTGCCGCATCGAATAAACGGCCTTCAACGTTTCTC
AATGTAGTGGCGCTAGGCAATACTGGTGCTGGTAAATCAGCTGTATTGAACAGTCTTCTA
GGACATCCTGCTTTGCCAACTGGTGAAGGAGGTGCTACTCGTGCTCCTATATGTATTGAA
CTTAAAAAGGATAGTTCCTTAAACAGCAAGTCAATTATCTTGCAAATCGACAGTAAATCC
CAACAAGTCTCTGCAAGTGCTCTTCGCCATTCTTTACAGGATAGACTAAGCAAGATCTCA
AACAAAAGCCGAGACGAGATATATTTGAAGCTTCGAACTAGTACAGATAGTTTTCTACTT
ATCTTTGTTGTTTTTGTGGTTTTACCTTGCTGTACAGCTCCTCCATTGAAGTTGGTTGAT
CTACCTGGAGTGGATAAGGGACATATTGATGATGCATTGAGTACATATGTTGCGCGCAGT
GATGCCATATTACTAGTGGTTATTCCCGCTGCTCTAGCACCAGAAATTTCCTCGTATAAA
GCACTTCGACTTGTGAAGGAGCATGATGGAGAATGTACAAGAACTATAGGTATTATTAGC
AAGGTAGATCAAGCAGCTTCAGATCCAAAAGTTCTTGCAGCTATCCATGCGCTTTTGCTA
AACCAGGGACCACCAAGCACATCCGATATCCCATGGGTTGCTTTGATTGGTCAATCTGTT
TCTATAGCTTCAGCCCAATCAGGAAATGTAGGCAATGATAATTCACTAGAAACAGCATGG
CGTGCTGAGAGTGAAAGTCTTAAATCAATATTGACAAAGGCTCCTCAAAGCAAGCTTGGT
AGGGTAGCATTAGTGGAGGTCCTTGCTCAACAGATCCGTAATCGAATGAAAGTCAGACTT
CCAAATCTTCTCTCAGGGCTCCAGGGAAAATCTCAATCAGTAAAGGATGAACTGGTAAAG
TTTGGTGATCAAATGGTTAATAGTGGTGAAGGTACAAAAGCTTTAGCCCTTGAGCTTTGT
CGTGAATTCGAGGATAAGTTTCTGGAACATCTTACAACTGGAGAGGGCGGTGGTTGGAAA
GTAATTGCAAGTTTTGAAGGCAAATTCCCTGATAGGATTAAGCAGCTACCTATGGATAGA
CACTTTGAGTTAAAAAATGTGAAGAGGGTTGTGCTGGAAGCAGATGGCTACCAACCTTAC
CTTATTTCTCCGGAGAAAGGGTTAAGGTCTTTAATAAAGACTGTGTTGGAGCTGGCGAAA
GAACCTTCAACACTTTGTGTCGAAGAGGTGATCTCTATCCGATGTTATTCTGCTTTATCA
AGGCAGTTAAATTATGCAATTTTGGCGGTACACAATTGTCTTTCAGAAGCATTTTGTTTT
TTTCTTCTCCTTTCAATACCTTGGCTGGCCTTTGGCAATACACTCATGAAAAATGAACCC
AACTTAATCAGAAATGTTGCATTTTTGGCACTATACAGTTGTCTTGCAGAAGTAATTGCA
ATTGCGACTACTGCTCTGGATGGGTTTAGAACTGAAGCAAAGAATATGGTTATTGCACTT
GTTGACATGGAGAGGGTTTATGTTCCCCCACAACACTTTATCCGTTTGGTGCAAAGGCGG
ATGGATAGGCAGCGCCGTGACGACGGCTTGAAGAACCAATCCTCCAAGAAGGCAGCACAG
GCAGAGCAATCCATGTTGACTAGGGCAACCAGCTCGCAGGCTGGAGATGAAAAAAGTAGT
TCAAAGTCGGGGAAGGATAAATCAGCTCAGCAAGATAAGGATTCACAAGAAGGACCAGTT
TTGAAGACTGCAGGACCCGATGGGGAGATAACAGCAGGGTTTCTACTGAAGAAAAGTGAT
AAAAAAAGTGGCTGGAGCAAGCGATGGTTTGTTTTAAATGACAAGACTGGAAAGCTTGGA
TATACCAAGAAGCAAGAAGAACGGCATTTCCATGGTGTAATTACTTTGGAGGAATGTAAT
CTTGAAGATGCTTCTGAAGAAGACGAACCATCAAAAAGTTCGAAAGATAAGAAGGCAACT
TGGCCTGATGGAGGAAAGGGACCTAGTCTCCTGTTCAAATTAACAAACAGGGTTCAATAC
AAGACTATTCTTAAAGGTGCCCAAAGTACTGTTATTTTGAAGGCAGAAACTTTAGCTGAG
AAGACTGAGTGGTTAAATAAATTAAAAAATGTCATAAGTTCTAAAGGAGGTCAAGTTATT
GCTGAATCCAGTCAACCTATGAGGCCAAGTTTATCAGAGGGGACACCTGGGACACCTGAC
ATGATGACTAGGAAACCAGCAGATCCAGAAGAAGAACTTCGATGGATGGCTCAAGAAGTA

CGTGGTTATGTTGAAGCTGTTCTCAACAGTCTTGCTGCTAACGTACCAAAGGCAGTTGTT
CTTTGCCAAGTAGAGAAGGCCAGAGAAGATATGCTTAATAAGTTGTACAGTTCTGTCAGC
GCACAAAGTAGAGCAAAGATCGAAGAGCTACTCCTGGAAGACCATAATGTGAAGCGTAGG
AGGGAGCACTTTCAGAAACAGTCCTCTCTTCTTGCTAAGGTCACTCAGCAGCTTAGTGTT
CATGATAATCGAGCAGCTGCTGCGTCTAGCTACTCAAATAGTGATGGAGCGGAAAGCGTC
TCAAGATCTGGTGGACAGTCATCAGGTGATGAATGGCGATCTGCATTTGATGGTACTTCA
AGTGCCCCTCAAAATGGTGATGCAGGGTCTAGAAGTCGTCGTACACCTAGCCGGATGCCA
CCTGCACCTCCTGGTTCTGGACAAAAATCCTAG
>FGENESH: 2 22 exon (s) 8226 - 20660 950 aa, chain +
MEAIEELEQLGDAMRQAAALLADEDVNEAAASNKRPSTFLNVVALGNTGAGKSAVLNSLL
GHPALPTGEGGATRAPICIELKKDSSLNSKSIILQIDSKSQQVSASALRHSLQDRLSKIS
NKSRDEIYLKLRTSTDSFLLIFVVFVVLPCCTAPPLKLVDLPGVDKGHIDDALSTYVARS
DAILLVVIPAALAPEISSYKALRLVKEHDGECTRTIGIISKVDQAASDPKVLAAIHALLL
NQGPPSTSDIPWVALIGQSVSIASAQSGNVGNDNSLETAWRAESESLKSILTKAPQSKLG
RVALVEVLAQQIRNRMKVRLPNLLSGLQGKSQSVKDELVKFGDQMVNSGEGTKALALELC
REFEDKFLEHLTTGEGGGWKVIASFEGKFPDRIKQLPMDRHFELKNVKRVVLEADGYQPY
LISPEKGLRSLIKTVLELAKEPSTLCVEEVISIRCYSALSRQLNYAILAVHNCLSEAFCF
FLLLSIPWLAFGNTLMKNEPNLIRNVAFLALYSCLAEVIAIATTALDGFRTEAKNMVIAL
VDMERVYVPPQHFIRLVQRRMDRQRRDDGLKNQSSKKAAQAEQSMLTRATSSQAGDEKSS
SKSGKDKSAQQDKDSQEGPVLKTAGPDGEITAGFLLKKSDKKSGWSKRWFVLNDKTGKLG
YTKKQEERHFHGVITLEECNLEDASEEDEPSKSSKDKKATWPDGGKGPSLLFKLTNRVQY
KTILKGAQSTVILKAETLAEKTEWLNKLKNVISSKGGQVIAESSQPMRPSLSEGTPGTPD
MMTRKPADPEEELRWMAQEVRGYVEAVLNSLAANVPKAVVLCQVEKAREDMLNKLYSSVS
AQSRAKIEELLLEDHNVKRRREHFQKQSSLLAKVTQQLSVHDNRAAAASSYSNSDGAESV
SRSGGQSSGDEWRSAFDGTSSAPQNGDAGSRSRRTPSRMPPAPPGSGQKS
